# Unraveling the role of the secretor antigen in human rotavirus attachment to histo-blood group antigens

**DOI:** 10.1371/journal.ppat.1007865

**Published:** 2019-06-21

**Authors:** Roberto Gozalbo-Rovira, J. Rafael Ciges-Tomas, Susana Vila-Vicent, Javier Buesa, Cristina Santiso-Bellón, Vicente Monedero, María J. Yebra, Alberto Marina, Jesús Rodríguez-Díaz

**Affiliations:** 1 Departament of Microbiology, Faculty of Medicine, University of Valencia, Valencia, Spain; 2 Department of Genomic and Proteomic, Instituto de Biomedicina de Valencia (IBV-CSIC) and CIBER de Enfermedades Raras (CIBERER), Valencia, Spain; 3 Department of Food Biotechnology, Institute of Agrochemistry and Food Technology (IATA-CSIC), Paterna, Spain; Institut Pasteur, FRANCE

## Abstract

Rotavirus is the leading agent causing acute gastroenteritis in young children, with the P[8] genotype accounting for more than 80% of infections in humans. The molecular bases for binding of the VP8* domain from P[8] VP4 spike protein to its cellular receptor, the secretory H type-1 antigen (Fuc-α1,2-Gal-β1,3-GlcNAc; H1), and to its precursor lacto-*N*-biose (Gal-β1,3-GlcNAc; LNB) have been determined. The resolution of P[8] VP8* crystal structures in complex with H1 antigen and LNB and site-directed mutagenesis experiments revealed that both glycans bind to the P[8] VP8* protein through a binding pocket shared with other members of the P[II] genogroup (i.e.: P[4], P[6] and P[19]). Our results show that the L-fucose moiety from H1 only displays indirect contacts with P[8] VP8*. However, the induced conformational changes in the LNB moiety increase the ligand affinity by two-fold, as measured by surface plasmon resonance (SPR), providing a molecular explanation for the different susceptibility to rotavirus infection between secretor and non-secretor individuals. The unexpected interaction of P[8] VP8* with LNB, a building block of type-1 human milk oligosaccharides, resulted in inhibition of rotavirus infection, highlighting the role and possible application of this disaccharide as an antiviral. While key amino acids in the H1/LNB binding pocket were highly conserved in members of the P[II] genogroup, differences were found in ligand affinities among distinct P[8] genetic lineages. The variation in affinities were explained by subtle structural differences induced by amino acid changes in the vicinity of the binding pocket, providing a fine-tuning mechanism for glycan binding in P[8] rotavirus.

## Author summary

The interaction of viruses with host glycans has become an important topic in the study of enteric virus infectivity. This interaction modulates several aspects of the viral cycle, including viral attachment, which in most cases depends on the host glycobiology dictated by the secretor and Lewis genotypes. The capacity to synthesize secretory type-I antigen H (fucose-α1,2-galactose-β1,3-N-acetylglucosamine; H1) at the mucosae, dictated by the presence of one or two functional copies of the fucosyl-transferase FUT2 gene (secretor status), has been clearly linked to infectivity in important enteric viruses such as the noroviruses. However, a big controversy existed about the contribution of H1 antigen to infection in the leading cause of viral gastroenteritis in young children (rotavirus). It has not been until recently that epidemiological data evidenced a diminished incidence of rotavirus in non-secretor individuals unable to produce H1. In the present manuscript we offer the evidence that P[8] RV bind H1 via a binding site common for the P[II] RV genogroup and that the H1 precursor lacto-*N*-biose (galactose-β1,3-*N*-acetylglucosamine; LNB) is also bound to this pocket with diminished affinity. The P[8] VP8* structures show a marginal role for the L-fucose moiety from H1 in protein interaction. However, its presence provides conformational changes in the LNB moiety that increase the affinity of VP8* for the H1 ligand and would account for a stronger RV binding to mucosa in individuals expressing H1 (secretors). We thus offer a mechanistic explanation for the different incidence of P[8] RV infection in different secretor phenotypes.

## Introduction

Rotaviruses are the leading etiologic agent of viral gastroenteritis in infants and young children worldwide and are responsible for an estimated 140,000 deaths each year in developing countries [1]. The typical classification of rotaviruses was derived from their genome composition and the immunological reactivity of three of their structural proteins: VP6, VP7 and VP4. Rotaviruses are classified into at least 7 groups (A to G) according to the immunological reactivity of the VP6 middle layer protein, with group A rotavirus being the most commonly associated with infections in human. The two outer capsid proteins VP7 and VP4, elicit neutralizing antibodies that can induce viral protection. Using these two proteins, a traditional dual classification system of group A rotaviruses into G (depending on the VP7 glycoprotein) and P (depending on the protease-sensitive VP4) types was established [2]. At least 36 different G-serotypes and 51 P-types have been identified among human and animal rotaviruses [3]. Viruses carrying G1[P8], G2[P4], G3[P8] and G4[P8] represent over 90% of human rotaviruses strains co-circulating in most countries [2], with the P[8] genotype, which comprises four different genetic lineages (I to IV [4]), being particularly relevant [5].

Diverse interactions between histo-blood group antigens (HBGAs) and rotavirus have been described and it is believed that HBGAs expressed on the surface of target cells serve as viral receptors. The distal VP8* portion (~27 kDa, N-terminal) of the rotavirus spike protein VP4 from P[8], P[4], P[6] and P[19] genotypes recognize the secretor HBGAs. P[8] and P[4] are closely related genetically and both genotypes were reported to bind the Lewis^b^ (Fuc-α1,2-Gal-β1,3-[Fuc-α1,4-]GlcNAc; Le^b^) and H type-1 (H1) antigens (Fuc-α1,2-Gal-β1,3-GlcNAc) by some authors [6], while there are controversial reports that show no Lewis^b^ binding for these genotypes [7]. P[6], a slightly further related genotype, binds the H1 antigen but not Lewis^b^ [6], whereas P[19] binds mucin core glycans with GlcNAc-β1,6-GalNAc motif and the type-1 HBGA precursor [8]. In addition, P[9], P[14] and P[25] genotypes bound specifically to the type A antigens (GalNAc-α1,3-[Fuc-α1,2-]Gal)[9, 10], whereas P[11] interacted with single and repeated *N*-acetyllactosamine (Gal-β1,4-GlcNAc; LacNAc), the type-2 precursor glycan [11]. Detailed evidences of VP8*-HBGAs interactions has been obtained by X-ray crystallography of P[14] VP8* and P[11] VP8* in complex with the type A oligosaccharide [9] and LacNAc [11], respectively. Recently, the structure of porcine P[19] VP8* complexed with lacto-*N*-fucopentaose I (Fuc-α1,2-Gal-β1,3-GlcNAc-β1,3-Gal-β1,4-Glc; LNFPI), and the mucin core-2 oligosaccharide (GlcNAc-β1,6-[Gal-β1,3] GalNAc) has been solved, showing a carbohydrate binding pocket alternative to the one used by P[11] and P[14] [7]. This binding pocket was first suggested by protein sequence analyses in other members belonging to the P[II] genogroup of rotaviruses (i.e. P[4], P[6], P[8] genotypes) [7] and recently confirmed for P[4] and P[6] VP8*s [12]. However, why non-secretors individuals (lacking α1,2 fucosylation in secretory HBGAs) have reduced rotavirus susceptibility [13–15] and what is the role of the secretory L-fucose in H1 ligand recognition for the most relevant human rotavirus remains unknown, as no structure was still available for the P[8] genotype in complex with ligand HBGAs. By using VP8* from a clinical isolate belonging to the lineage III of P[8], in the present work we show that P[8] VP8* binds H1 antigen at a similar site as their ligand HBGAs bind to P[19], P[4] and P[6] genotypes. Our structural and functional results also show that the H1 precursor lacto-*N*-biose (Gal-β1,3-GlcNAc; LNB), devoid of L-fucose, also interacts with VP8* and we discard Lewis^a^ or Lewis^b^ antigens as ligands of P[8] genotypes. We provide the molecular bases for the role of secretor antigen in rotavirus binding to its receptor. Our results show two-fold increase in the affinity for the H1 antigen compared to LNB. This increase is explained by reduced contacts of the L-fucose with solvent molecules and the structural stabilization of LNB moiety in the competent conformation for binding. Furthermore, we show how subtle differences at the H1/LNB binding pocket in different P[8] lineages influence antigen affinities, giving clues for the relevance of the host glycobiology in P[8] rotavirus impact in humans. The partial anti-adhesin effect of LNB against rotavirus reported in the present article and the acquired knowledge on rotavirus-host cells interaction during virus attachment might open new avenues for the treatment and prevention of rotavirus infections.

## Results

### The rotavirus VP8* domain from the P[8] genotype recognizes the H type-1 antigen precursor lacto-*N*-biose

**Fig 1:**
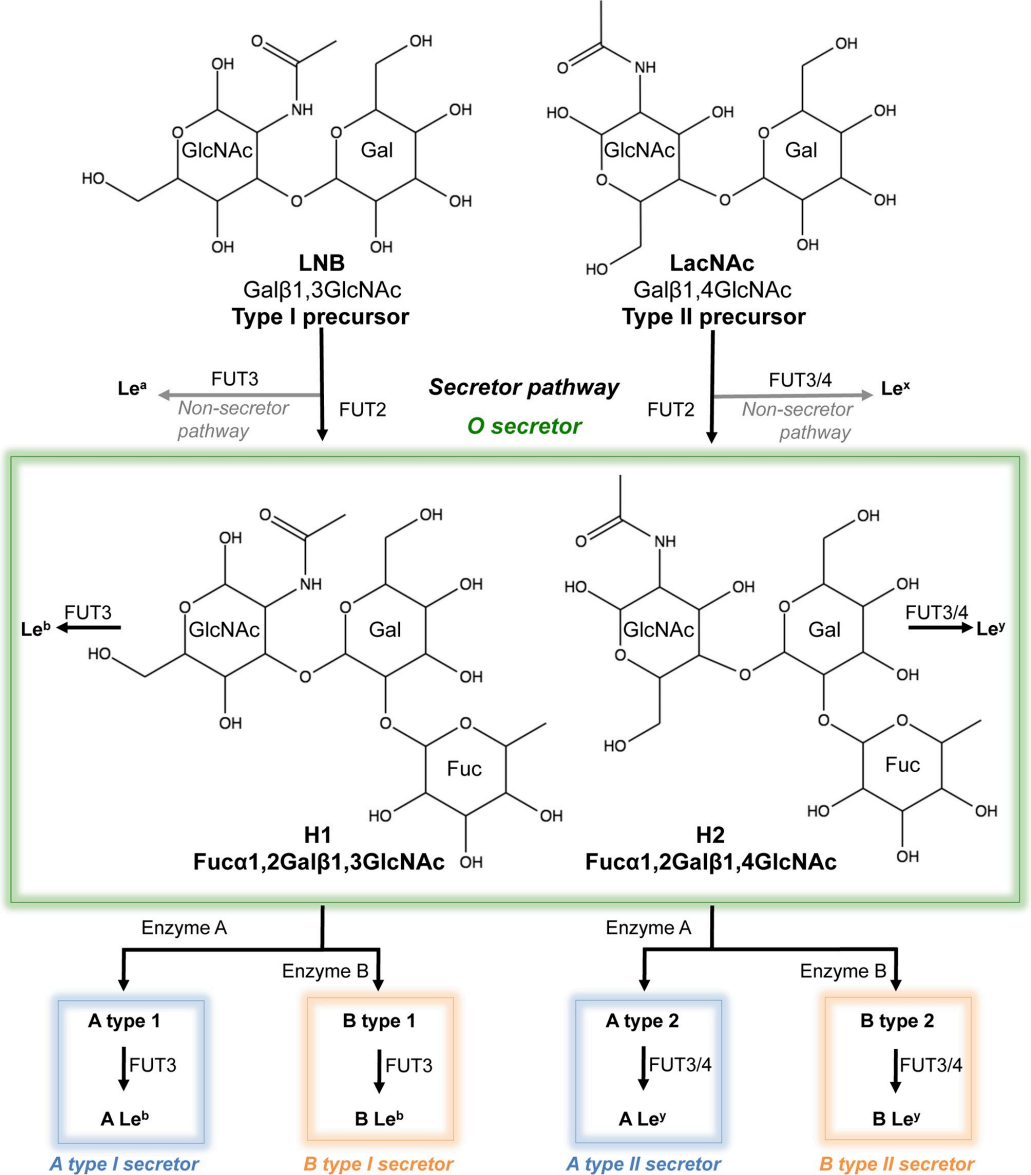
Structure and schematic representation of the biosynthetic pathways of human type 1 and 2 histo-blood group antigens (HBGAs). The structures the H1 and H2 antigens as well as their precursors LNB and LacNAc are shown.

**Fig 2:**
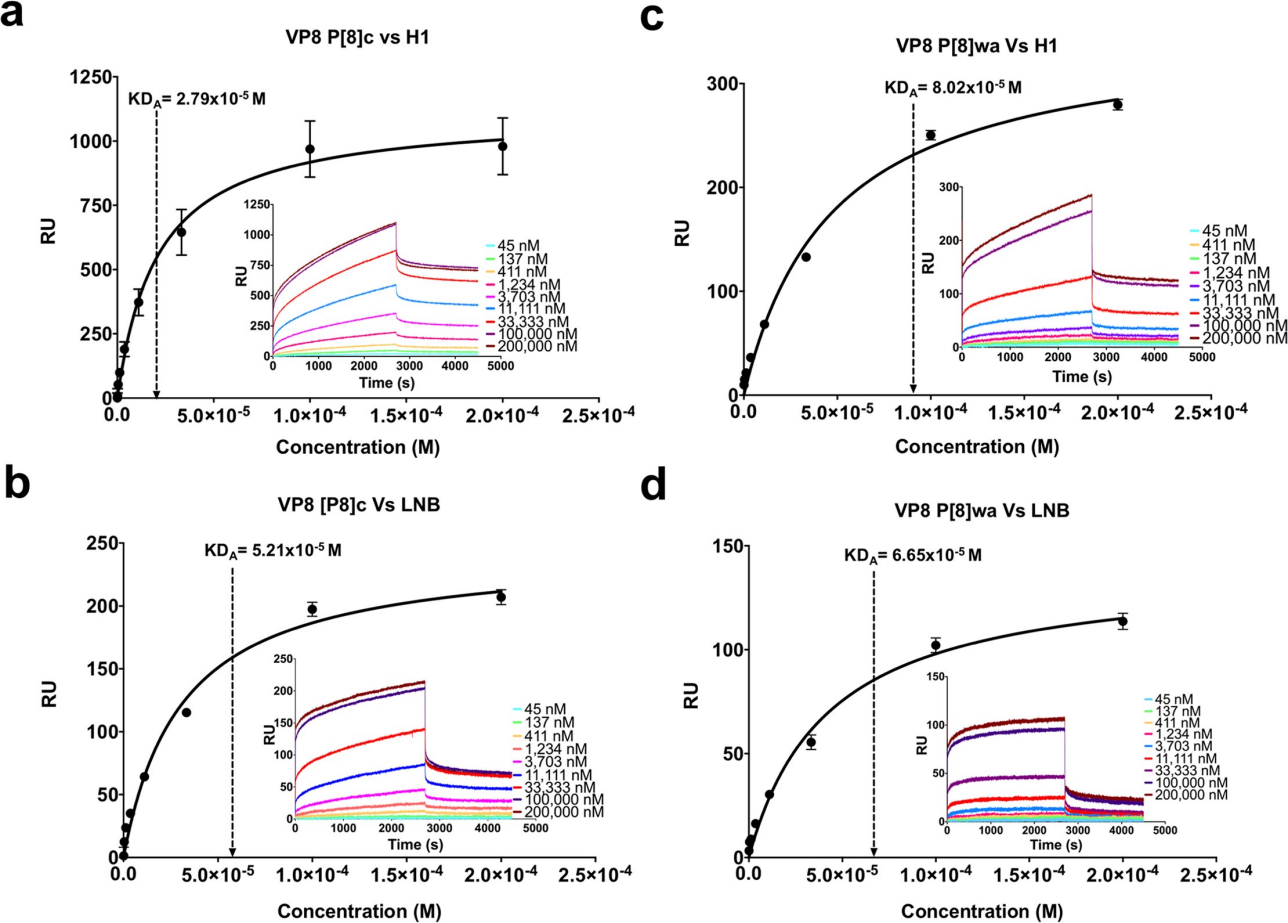
Binding characterization of the H1 antigen and its precursor to VP8* from the P[8] genotype. Panel **a** shows the affinity fit and sensorgrams of the interaction between P[8]_c_ and the H1 antigen obtained by SPR. Panel **b** shows the affinity fit and sensorgrams of the interaction between P[8]_c_ and LNB obtained by SPR. Panels **c** and **d** show the affinity fit and sensorgrams of the interactions between P[8]_Wa_ and the H1 antigen and P[8]_Wa_ and LNB obtained by SPR, respectively. The bars indicate the standard deviation.

VP8* domains from P[4], P[6], P[9], P[11], P[14], P[25] genotypes and from different genetic lineages (I, III and IV) from P[8] genotype were produced (S1 Fig). In order to confirm their functionality, the different proteins were challenged by an ELISA-like binding assay against a panel of biotinylated histo-blood group antigens (HBGAs) (Fig 1 and S1 Table), corroborating the previously described interactions (S2 Fig). Genotypes P[4], P[6] and P[8] recognized the H type-1 antigen (Fuc-α1,2-Gal-β1,3-GlcNAc, H1), P[11] recognized the H type-2 antigen (Fuc-α1,2-Gal-β1,4-GlcNAc, H2) and P[9], P[14] and P[25] the blood group A antigen trisaccharide (GlcNAc-α1,3-(Fucα1,2)-Gal, A_tri_). The P[8] genotype additionally displayed low binding to this trisaccharide. However, and contrarily to previous reports [7], in our assays genotypes P[4] and P[8] exhibited very low or absence of binding to Lewis^b^ (Fuc-α1,2-Gal-β1,3-[Fuc-α1,4-]GlcNAc). Remarkably, VP8* from P[8] genotype recognize the H1 antigen precursor lacto-*N*-biose (Gal-β1,3-GlcNAc, Lewis^c^, LNB; S2 Fig) but differences in the binding abilities were found among different genetic lineages and strains. Thus, VP8* from the cultivable human rotavirus Wa (P[8]_Wa_) and the Rotarix vaccine (P[8]_Rotarix_) strains (both lineage I) gave lower signals in the ELISA tests with H1 and LNB than the VP8* from the lineage IV strain (P[8]_LIV_) and from a clinical isolate belonging to lineage III (P[8]_c_) (S2 Fig). The newly discovered interaction with LNB was further characterized by testing different concentrations of VP8* from this isolate (P[8]_c_) and the cultivable P[8]_Wa_ (S3 Fig). The results showed that both proteins were able to bind H1 and LNB, although binding to the first antigen was higher. We performed a more detailed characterization of the interaction of VP8* to H1 and LNB by determining the apparent affinity constants (Kd_a_) for each interacting pair by SPR (Table 1). The Kd_a_ of the VP8* P[8]_c_ recognition of the H1 antigen (Kd_a_ = 27.9 ± 0.71 μM; Fig 2A) was two-fold lower compared to the LNB precursor (Kd_a_ = 52.1 ± 4.26 μM; Fig 2B), this difference was significant (*p* = 0.0045) suggesting that the H1 L-fucose moiety contributes actively to the binding. Surprisingly, the affinity constant for the interaction of VP8* from P[8]_Wa_ with H1 was three times higher (Kd_a_ = 80.2 ± 2.21 μM) than that of P[8]_c_ (Fig 2C). Furthermore, the VP8* from P[8]_Wa_ showed a similar apparent affinity for LNB than for H1 (Kd_a_ = 66.5 ± 6.47 μM; *p* > 0.05 Fig 2D). Interestingly, interactions for VP8* from Rotarix strain (lineage I) and the lineage IV strain with H1 and LNB were too low to be determined under our SPR conditions.

**Table 1:** Affinity constants between different VP8* proteins and HBGAs determined by SPR.

Analyte vs Ligand	Kd_a_ (M)^a^
P[8]_c_ vs H1	2.79 x 10^−5^ ± 7.17 x 10^−7^
P[8]_c_ vs LNB	5.21 x 10^−5^ ± 4.26 x 10^−6^
P[8]_Wa_ vs H1	8.02 x 10^−5^ ± 2.21 x 10^−6^
P[8]_Wa_ vs LNB	6.65 x 10^−5^ ± 6.47 x 10^−6^
P[8]_c_ M4(Ile173Val) vs H1	3.90 x 10^−5^ ± 1.25 x 10^−6^
P[8]_c_ M4(Ile173Val) vs LNB	4.94 x 10^−5^ ± 1.74 x 10^−6^

^a^ Apparent affinity constant ± the standard deviation

**Fig 3:**
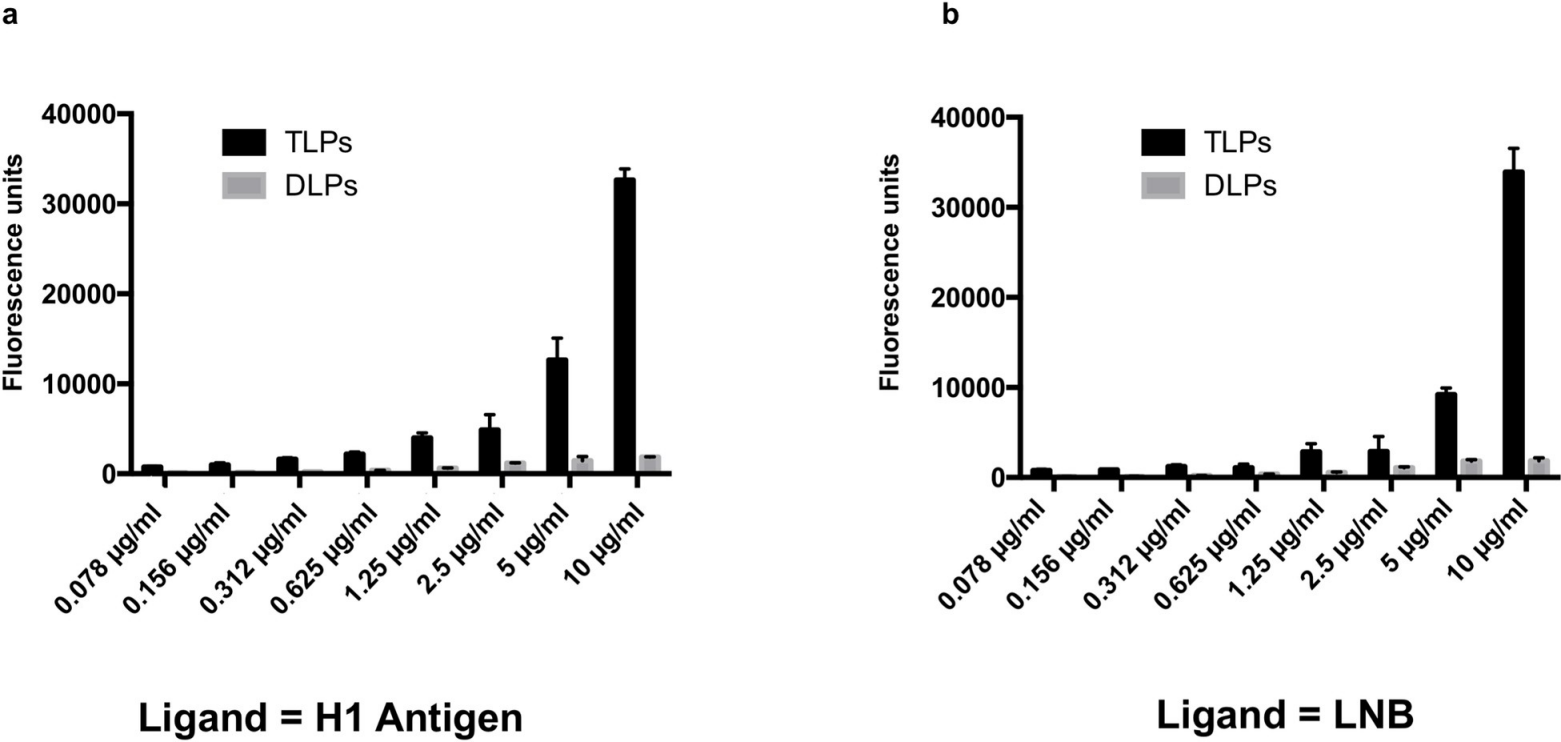
Binding of rotavirus particles to the H1 antigen and to lacto-*N*-biose (LNB). Rotavirus triple layered particles (TLPs) and double layered particles (DLPs) were used in binding assays against the H1 antigen (panel **a**) and against its precursor (LNB) (panel **b**).

To further characterize the role of VP8* interaction with the H1 precursor LNB, complete virions of the Wa strain (triple-layered particles; TLP) and double-layered particles (DLP; obtained after VP4 and VP7 removal by EDTA treatment) were assayed by an ELISA-like binding assay. The Wa TLP, but not the Wa DLP, were able to bind H1 antigen (Fig 3A) and its precursor LNB (Fig 3B), in a concentration-dependent manner. This indicated that the observed interaction between P[8] VP8* and LNB was also relevant in a complete rotavirus context. These results also point to the fact that despite the low affinity of P[8] VP8* from lineage I to H1 and LNB, the high avidity of a multi-binder particle (virions contain 120 molecules of VP4) results in a measurable interaction.

### Soluble lacto-*N*-biose and galacto-*N*-biose partially block the binding of VP8* to H-type 1 antigen and to its precursor

**Fig 4:**
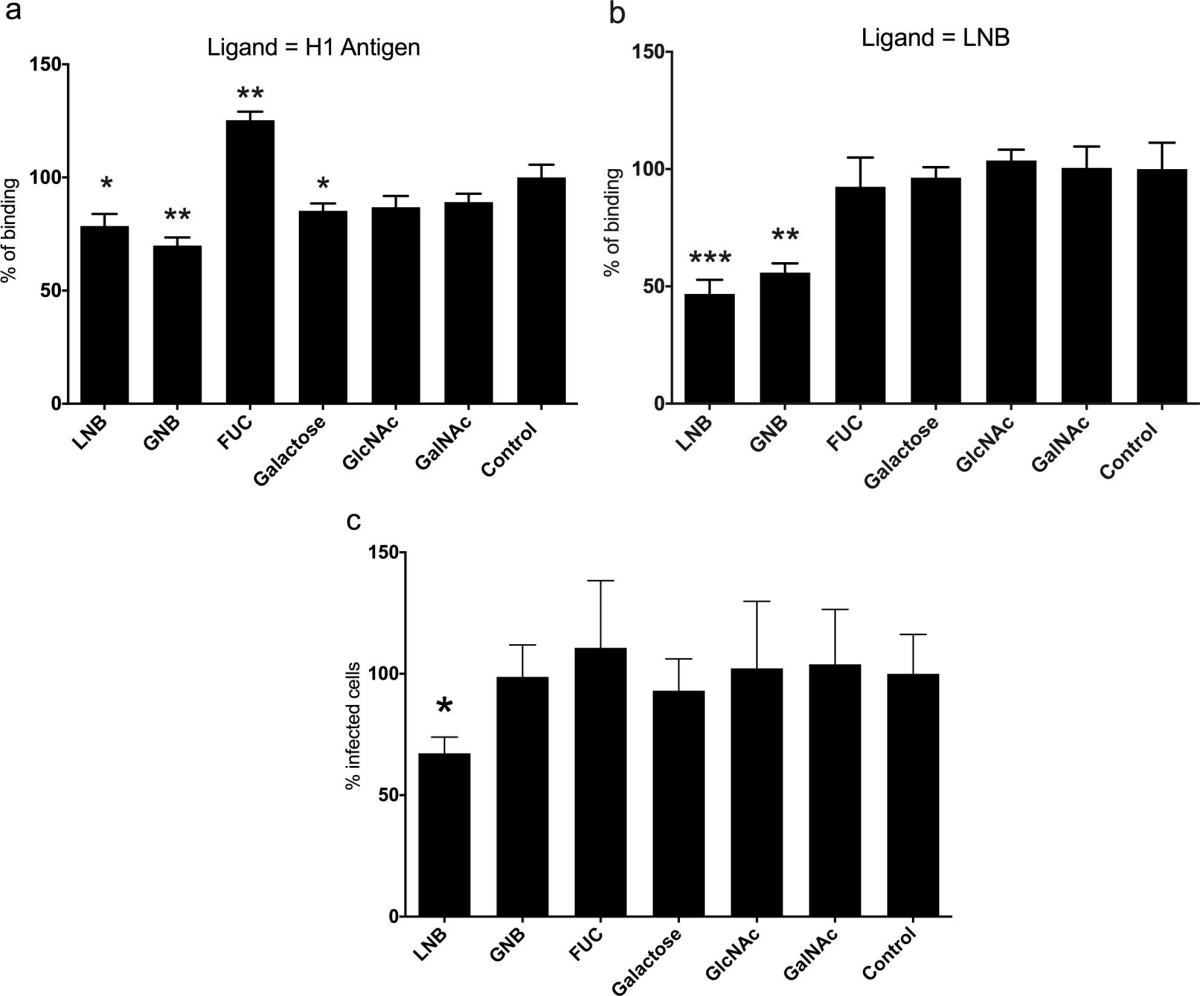
Binding and infection blocking experiments. Soluble lacto-*N*-biose (LNB) and galacto-*N*-biose (GNB) as well as the monosaccharides L-fucose (FUC), D-galactose, *N*-acetylglucosamine (GlcNAc) and *N*-acetylgalactosamine (GalNAc) were used at 20 mM to block the binding of VP8* from the P[8]_c_ genotype to the H1 antigen (panel **a**) and to LNB (panel **b**). The same sugars were utilized at 5 mg/mL to block the infection of Wa rotavirus in MA104 cells. Statistical differences are indicated with asterisks. * *p* < 0.05; **p < 0.01; ***p = 0.001 (panel **c**).

To confirm the binding of VP8* from the P[8]_c_ genotype to LNB we settled up a binding blocking assay where LNB (Gal-β1,3-GlcNAc) and its structurally-related disaccharide galacto-*N*-biose (Gal-β1,3-GalNAc; GNB), were tested as potential inhibitors of binding. The results showed a moderate but significant (*p* < 0.05) reduction in the binding to the H1 antigen by both disaccharides (24.2% reduction for LNB and 30.1% for GNB; Fig 4A). The monosaccharide constituents of LNB and GNB (D-galactose, *N*-acetyl-glucosamine and *N*-acetyl-galactosamine) and L-fucose were also tested (Fig 4). Among these sugars only D-galactose possessed a discrete but significant blocking capacity of VP8* binding to the H1 antigen (14.7% reduction; *p* = 0.032). Interestingly, soluble L-fucose significantly increased the binding of VP8* to the H1 antigen (Fig 4A). As expected, when the precursor LNB was used as the ligand, soluble LNB and GNB were also able to reduce the binding of VP8* P[8]_c_, by 52.2% and 44.1%, respectively (Fig 4B).

We next investigated the role of the H1 precursor antigen in rotavirus infection by incubating MA104 cells with rotavirus Wa strain that was preincubated with LNB, GNB, their monosaccharide constituents and L-fucose. Only LNB significantly blocked viral infection (33% reduction; Fig 4C), suggesting that this HBGA precursor interferes with the binding of the rotavirus Wa strain to its receptor in MA104 cells.

### Glycans bind VP8* in a preformed binding pocket

**Fig 5:**
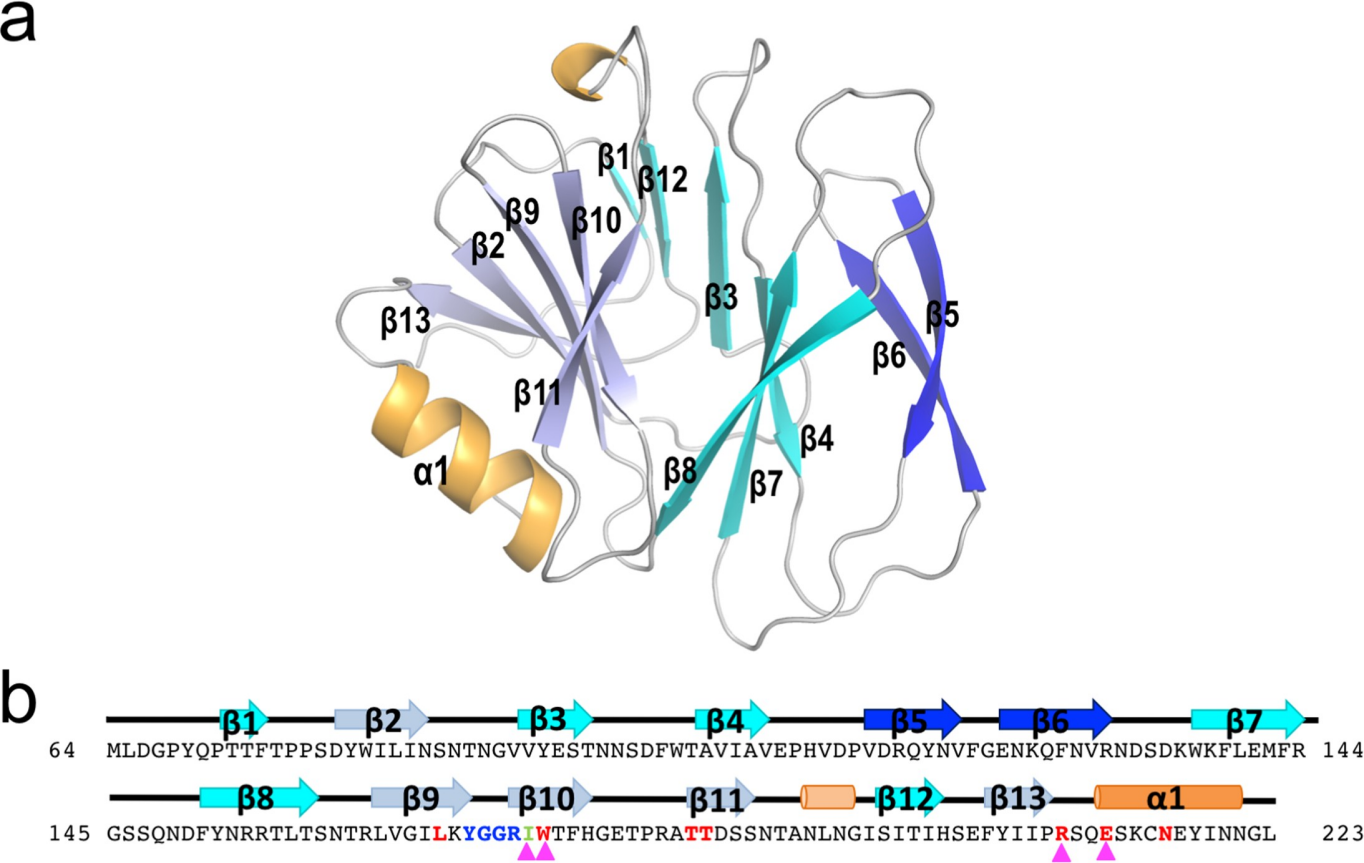
Structure of P[8]_c_ VP8*. **a** Cartoon representation of P[8]_c_ VP8* Apo1 with the secondary structural element are colored in blue for β-sheets, orange for α-helices and white for loops. Different tones of blue are used to differentiate among β-strands. Secondary structural element are numbered and labeled in order from the N to C terminus. The sequence of P[8]_c_ VP8* is shown in panel **b**, highlighting with red lettering the residues that interact with H1 sugar and with blue lettering the β9-β10 hairpin. Magenta triangles indicate residues mutated in this study. Structural elements are shown above the sequence colored as in the cartoon representation.

**Fig 6:**
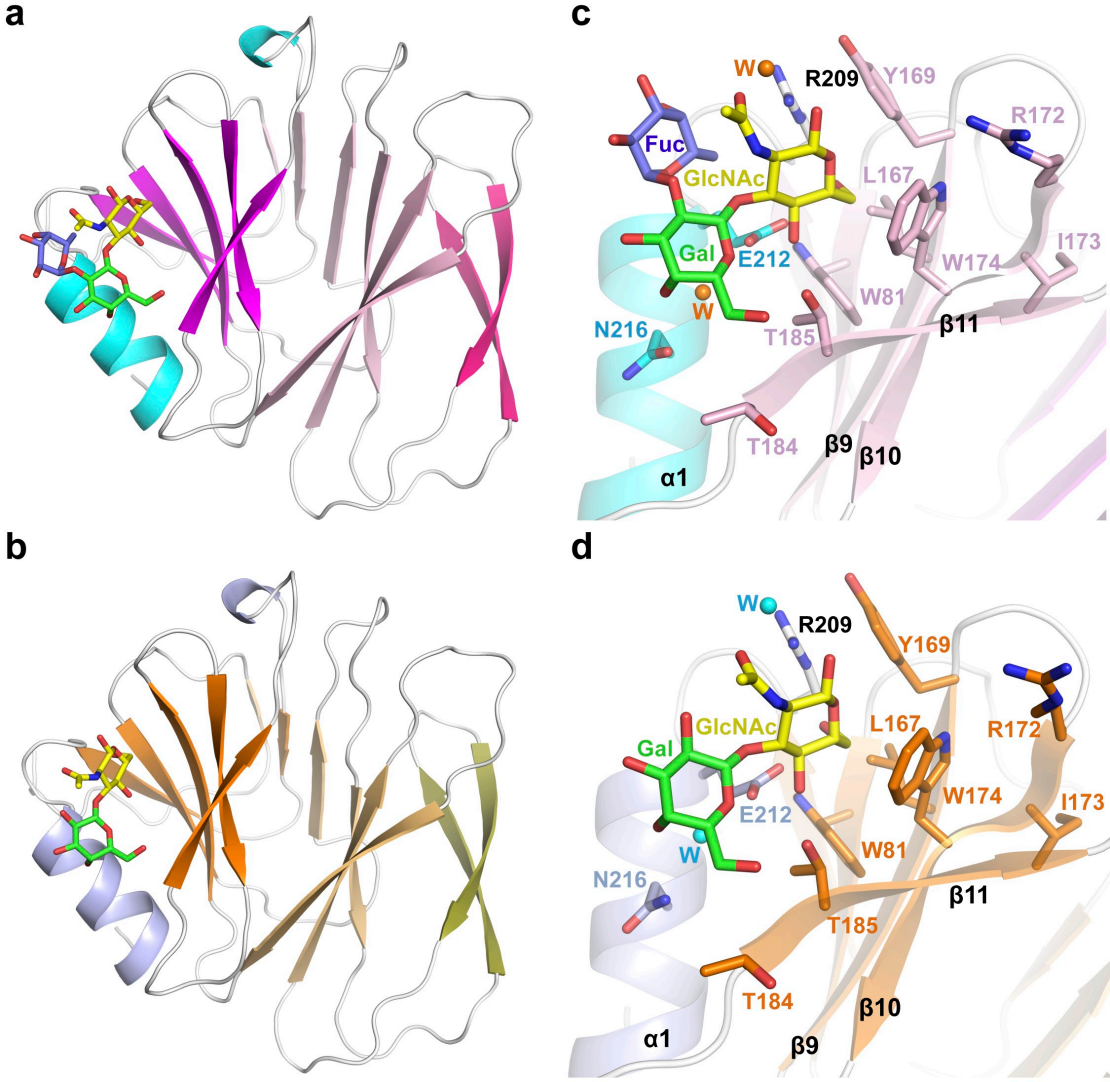
Structures of P[8]_c_ VP8* in complex with H1 antigen and LNB. **a** Cartoon representation of P[8]_c_ VP8* H1 structure with β-strands in different pink tones to differentiate among β-sheets, α-helices in cyan and loops in white. The H1 antigen bound between a β-sheet and the α-helix is shown in stick with carbon atoms in yellow, green and slate blue for the GlcNAc, Gal and Fuc motives. **b** Cartoon representation of P[8]_c_ VP8* LNB structure in identical orientation with β-sheets in different orange tones and α-helices in light-blue. The bound LNB is represented as in (a). **c** and **d** Close view of the P[8]_c_ VP8* H1 (**c**) and P[8]_c_ VP8* LNB (**d**) active centers. The bound sugars are represented in sticks with atoms in yellow (GlcNAc), green (Gal) and slate blue (Fuc). The residues interacting with the sugars are shown in stick representation, with carbon atoms colored according to the structural element to which they correspond (semi-transparent) and are labeled. Nitrogen and oxygen are colored in dark blue and red, respectively. Waters interacting with the sugars are represented as orange (**c**) and cyan (**d**) spheres. The mutated I173 in the back side of the sugar binding pocket is shown as stick in both panels.

To understand the molecular basis of LNB and H1 recognition and binding to P[8] VP8* we determined the crystal structure of the clinical isolate P[8]_c_ VP8* in its apo form and bound to LNB and H1 glycans. Two different crystalline forms of P[8]_c_ VP8* in its apo form were obtained. The first form, VP8*-Apo1, diffracts at 1.35 Å resolution and presents a single copy of P[8]_c_ VP8* in the crystal asymmetric unit (ASU) while the second form, VP8*-Apo2, diffracts to 1.5 Å and presents two copies in the ASU (Table 2). The three copies of P[8]_c_ VP8* in these two crystalline forms present the galectin fold with two twisted β-sheets separated by a superficial cleft that conforms the glycan binding site in P[11] and P[14] genotypes [9, 16] (Fig 5). Superimposition of the individual VP8* protomers from these crystals showed that the three molecules of P[8]_c_ VP8* are almost identical (RMSD 0.28 Å for the superposition of all the Cα; S4A Fig and S2 Table). Crystals of P[8]_c_ VP8* bound to H1 or its precursor LNB were obtained in a third different crystalline form and the structures of P[8]_c_ VP8*-H1 and P[8]_c_ VP8*-LNB complexes were solved to 1.8 and 1.3 Å, respectively (Fig 6A, 6B and Table 2). Two protomers of VP8* were present in the ASU of each of these crystals and, remarkably, only one out of the two protomers showed a bound glycan molecule. Sugar binding induces negligible conformational changes in the VP8* (S4A Fig) since the structural comparison of the glycan-bounded and glycan-free P[8]_c_ VP8* protomers showed minimal differences (RMSD 0.22–0.35 Å; S2 Table). Furthermore, P[8]_c_ VP8* are also structurally identical (RMSDs 0.37–0.57 Å) to the VP8* apo forms of the linage I P[8]_Wa_ and P[8]_Rotarix_ (S4B Fig and S2 Table), supporting that the glycan binding site is preformed in the P[8]_c_ VP8* protein. This characteristic also seems to be shared by other P genotypes belonging to the P[II] genogroup, since structural comparison with recently reported glycan-bound structures of VP8* from human P[4] and P[6], and porcine P[19] genotypes showed modest differences (RMSD 0.51–0.84 Å) (S4C Table and S2 Table).

**Table 2:** Data collection and refinement statistics.

	P[8]_C_ VP8* Apo1	P[8]_C_ VP8* Apo2	P[8]_C_ VP8* LNB	P[8]_C_ VP8* H1
**Data Collection**				
Beamline	ALBA-XALOC	ALBA-XALOC	ALBA-XALOC	ALBA-XALOC
Wavelength (Å)	0.97910	0.97907	0.97923	0.97928
Space group	P3_2_ 2	P 2_1_ 2_1_ 2_1_	P2_1_	P2_1_
Cell dimensions (Å)	a = b = 76.15 c = 70.08 α = γ = 90 β = 120	a = 53.35 b = 77.74 c = 104.01 α = β = γ = 90	a = 38.99 b = 54.72 c = 67.43 α = γ = 90 β = 97.92	a = 38.74 b = 54.64 c = 67.94 α = γ = 90 β = 97.73
Resolution (Å)	65.95–1.35 (1.39–1.35) ^a^	104.01–1.51 (1.59–1.51)	54.72–1.31 (1.38–1.31)	54.64–1.85 (1.95–1.85)
Total reflections	524841 (27293)	483220 (61018)	220889 (26591)	85661 (12054)
Unique reflections	52269 (5152)	68175 (9712)	64030 (9582)	23811 (3447)
Completeness (%)	99.92 (99.34)	99.8 (98.9)	94.9 (97.6)	99.2 (98.9)
Multiplicity	10.0 (5.3)	7.1 (6.3)	3.4 (2.8)	3.6 (3.5)
Mean I/σ(I)	15.98 (1.48)	10.2 (2.3)	8.4 (1.7)	9.5 (1.9)
Rpim	0.021 (0.266)	0.051 (0.425)	0.053 (0.419)	0.053 (0.431)
CC 1/2	0.999 (0.932)	0.996 (0.802)	0.995 (0.739)	0.997 (0.746)
**Refinement**				
R_work_	0.206	0.171	0.180	0.176
R_free_	0.227	0.204	0.209	0.232
Number of atoms	1564	3202	3204	2819
Protein	1312	2649	2652	2604
Water	211	524	512	179
Others	41	29	40	36
Rmsd, bonds (Å)	0.032	0.022	0.022	0.016
Rmsd, angles (°)	2.703	2.224	2.105	1.717
**Ramachandran plot**				
Preferred (%)	94.77	97.04	97.41	95.91
Allowed (%)	4.58	2.96	2.59	3.77
Outliers (%)	0.65	0	0	0.31

^a^Numbers in parentheses indicate values for the highest-resolution cell.

### P[8] glycan binding site

P[8]_c_ VP8* binds H1 and LNB in a pocket formed by one of the β-sheets and the C-terminal α helix (Fig 6A and 6B). The structures showed that the LNB moiety is embedded in the pocked interacting with the protein while the L-fucose is projected out with minimal, mainly mediated by solvent, contacts with the protein (Fig 6A and 6B). Only seven residues recognize the LNB, contacting the *N*-acetyl-glucosamine moiety via L167, W174, T185, R209, and E212 and the galactose moiety via T184, T185, E212 and N216 (Fig 6C, 6D and S3 Table). This pocket shared identical interacting residues to the novel VP8* binding pocket recently discovered in P[19] [7] and P[4]/P[6] [12] genotypes for their interaction with LNFPI and it differs from the previously defined carbohydrate binding site in VP8* from P[11] (LacNAc binding [11]) and P[14] (A-antigen binding [9]) genotypes, that is located in the cleft between the two β sheets (S4D Fig). The *N*-acetyl-glucosamine has a major contribution to the binding since it is inserted in the pocket while the galactose acquires a more superficial position (Fig 6C and 6D). In this way, the *N*-acetyl-glucosamine ring is packet between W174 and R209 that define two faces of the binding site and the O4 and O6 oxygens mediated hydrogen-bonds with E212 that it placed at the bottom of the pocket (Fig 6C, 6D and S2 Table). To confirm the role of these amino acids in H1 and LNB interaction, the P[8]_c_ VP8* mutants W174A (M1, VP8^W174A^), R209A (M2, VP8^R209A^) and E212A (M3, VP8^E212A^) were obtained (S1 Fig). The three mutant VP8* lost their ability to interact with their receptor when they were assayed for binding to H1 and LNB by SPR (S5 Fig), supporting the structural data and suggesting that the identified pocket is the only binding site for H1 antigen and its precursor in P[8] VP8*.

### Lacto-*N*-biose fucosylation and P[8] VP8* binding

**Fig 7:**
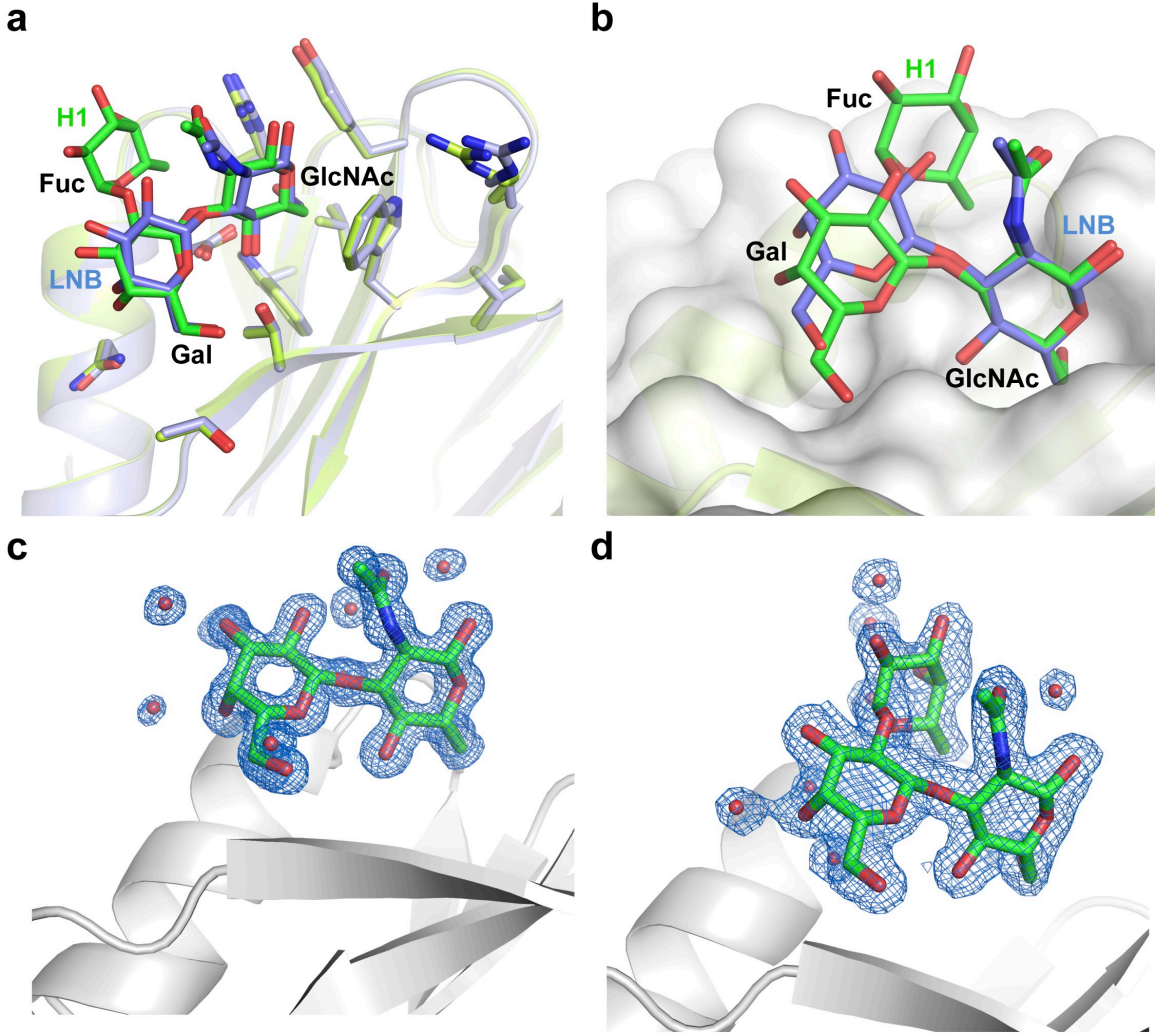
Comparison of H1 and LNB sugar binding by P[8]c VP8*. **a** Close view of the glycan binding site of superimposed P[8]c VP8* H1 (light green) and P[8]c VP8* LNB (light blue) structures (in semitransparent cartoon) showed that sugar interacting residues (in sticks and colored according to the corresponding structure) are disposed with minimal differences in comparison with the corresponding bound sugars (in sticks and colored green and stale blue for H1 and LNB, respectively). **b** Superimposition of the GlcNAc moieties of the H1 (in stick colored with carbons in green) and LNB (in stick colored with carbons in stale blue) from the structures of its complexes with P[8]c VP8* shows that the galactose moiety in the LNB structure occupies a more shallow position. **c** and **d** The high quality electron density maps (2Fo—Fc contoured in blue at 1σ) obtained by X-ray diffraction at 1.3 and 1.85 Å resolution for P[8]c VP8* in complex with LNB (**c**) and H1 (**d**), respectively, provides a high level of details as shown for the modelled ligands represented in sticks (carbons, oxygen and nitrogen colored in green, red and blue, respectively) and the coordinated molecules of solvent represented as red spheres inside the maps. P[8]_c_ VP8* proteins are shown as grey cartoons.

Fucosylation of the H1 precursor is genetically determined by the FUT2 gene (α1,2 fucosylation of the terminal galactose), resulting in different secretor status and by the FUT3 gene (α1,4 fucosylation at the precursor *N*-acetyl-glucosamine), defining the Lewis status (Fig 1). This genetically determined glycan profile is a susceptibility factor in human rotavirus [17, 18]. Our P[8]c VP8* structure in complex with H1 antigen confirms that the secretory L-fucose portion has reduced contacts with VP8* that are mediated by solvent molecules. Similarly, minimal or null contacts of the L-fucose moiety were observed in the complexes of LNFPI with other VP8* from the P[II] genogroup [7, 12]. Therefore, the difference in the affinity for H1 and LNB in diverse VP8* from this genotype were difficult to explain. A close view of the relative disposition of the precursor LNB moiety in both P[8]_c_ VP8* complexes after superimposition of VP8* proteins showed some differences but not so for the interacting residues (Fig 7A). If, alternatively, the *N*-acetyl-glucosamine moieties from both structures are superimposed it becomes visible that the galactose moiety occupies a more solvent-exposed position in the LNB structure compared to H1 (Fig 7B), explaining its weaker binding in comparison with this antigen. The atomic resolution of the diffraction data and the high quality of the density maps derived from these data (Fig 7C and 7D) allows modeling accurately the ligand structures to observe these differences. This observation indicates that the L-fucose moiety induces an LNB conformation more suitable for the union to VP8*. The P[8]_c_ VP8* structure in complex with H1 shows that the L-fucose ring stacks over the acetamido group of *N*-acetyl-glucosamine stabilizing the glycan conformation (Figs 6C and 7B). Therefore, the structures suggest that the L-fucose moiety could favor the VP8* binding by a double mechanism: inducing a competent conformation that facilities the LNB module recognition and binding and mediating indirect interactions that stabilize the glycan-VP8* complex.

Oppositely, the fucosylation in the *N*-acetyl-glucosamine in the LNB precursor to produce the Lewis^b^ antigen seems to be incompatible with the binding to P[8] VP8*. Docking of the Lewis^b^ antigen (Fuc-α1,2-Gal-β1,3-[Fuc-α1,4-]GlcNAc) in the P[8]_c_ VP8* structure taking H1 antigen as a reference showed that the α1,4-linked L-fucose points towards the inside of the glycan binding pocket, clashing with different residues (mainly T185 and E212) that generate this cavity (S6A Fig). Since glycan binding pocket is highly conserved among P[8] VP8* linages and P[II] genotypes [7, 12], it seems that this group of rotaviruses are non-competent to bind Lewis HBGAs. This observation is confirmed by our ELISA assays where any of the VP8* proteins from P[II] genotype showed binding capacity to these HBGAs, and it questions previous results were P[8] VP8* was shown to interact with Lewis^b^ [6]. Following an identical approach the A-type I antigen can be docked in the P[8]_c_ VP8* sugar-binding pocket. The docked sugar showed that the *N*-acetylgalactosamine (GalNAc) added to the non-reducing end of the H1 galactose is pointing towards the solvent without showing any interaction with the protein (S6B Fig), supporting that P[8] VP8* should be able to bind this sugar.

### Subtle structural differences affect glycan binding affinity and specificity

The structures of VP8* in complex with LNB and H1 obtained here confirm that P[4], P[6], P[19] and the prevalent human genotype P[8] of rotavirus share a common glycan binding site which is highly conserved in structure and sequence. Therefore, the differences in affinity and specificity for HBGAs observed in our ELISA and SPR assays as well as those reported by others are striking. Particularly, the differences in the capacity to interact with the H1 antigen observed among P[8] lineages are difficult to understand. Inspection of the amino acids defining the glycan binding site in the four P[8] lineages oriented by the comparison of the P[8]_c_, P[8]_Wa_ and P[8]_Rotarix_ structures revealed interesting differences. First, the VP8* protein from Rotarix strain (lineage I) differed from other members of the same lineage, including the Wa strain, by the replacement of leucine 167 by phenylalanine. Since L167 is placed at the bottom of the sugar-binding pocket (Fig 6C and 6D), the introduction of a bulky Phe residue could explain the lack of interaction of this vaccine strain to H1 [19] that was corroborated in our study. Second, a close inspection of the P[8] structures revealed small differences in the disposition of the β-hairpin connecting the strands β9 and β10 (S7 Fig). These two β-strands define the bottom of the glycan binding pocket where the LNB moiety is settled (Fig 6C and 6D). These subtle movements are allowed by the presence of two Gly residues (G170 and G171) conferring flexibility to the loop. Y169 and R172 at both sides of the Glys delimit the loop and play a pivotal role in the correct organization of the binding pocket since Y169 stacks over R209 and R172 interacts with W174, the main residues recognizing *N*-acetyl-glucosamine (Fig 6C, 6D and S7 Fig). Therefore, the disposition of this loop could modulate de glycan affinity and specificity. The sequence analysis of this region among P[8] lineages reveals that at position 173 lineage I presents a Val whereas an Ile is found in the rest of lineages (S4 Table). Position 173 is placed in the base of the β-hairpin facing to the hydrophobic core of VP8* protein (Fig 6C, 6D and S7 Fig). Therefore, we wondered if this subtle change could influence the glycan-binding site architecture accounting for the difference in the H1 affinities observed between lineages. To test this possibility we designed a new mutant in which I173 in P[8]_c_ VP8* was replaced by Val (M4, VP8^I173V^), emulating P[8]_Wa_. This replacement resulted in a VP8* with a diminished interaction with the H1 antigen, with an affinity constant (Kd_a_ = 39.0 ± 1.25 μM, S5 Fig, Table 1) 1.4-fold higher compared to the wild-type protein (Kd_a_ = 27.9 ± 0.71 μM, Table 1; *p* = 0.0002). The VP8^I173V^ variant retained the binding ability to LNB with an apparent affinity constant (Kd_a_ = 49.4 ± 1.74 μM, Table 1) that was still higher than that of H1 (*p* = 0.0033). These results support that subtle amino acid changes at the loop close to the binding pocket may have contributed to modulate the glycan affinity between P[8] VP8* from lineages I and III. Differences at this site are also evident in the structures of VP8* from the P[II] genogroup. While conservation between P[8] and P[4] is high, the sequence divergence between P[4] and P[6] (S4 Table) might explain the different affinities for LNFPI between these two genotypes [12].

## Discussion

Virus-HBGAs interaction has emerged as an important factor in viral infectivity. Contrarily to other enteric viruses (i.e.: norovirus), the relevance of HBGA interaction in rotaviruses was first neglected, and virus-host cell attachment studies were mainly focused on binding to sialic acid, until interactions with HBGA were suggested by VP8* structural analyses [20] and experimentally determined in sialidase-insensitive strains [6]. In norovirus many studies point to the human FUT2 polymorphism as a key feature affecting viral infectivity [17, 18]. Individuals carrying two null FUT2 alleles lack fucosyl transferase-2 activity, do not express H antigen structures at the intestinal mucosa and in secretions (non-secretors) and are less susceptible to norovirus. While previous studies showed no correlation between the secretor status and rotavirus infection [17], the most recent studies show that antibody titers to rotavirus [13], rotavirus gastroenteritis incidence [14] and vaccine take [15] correlate with the FUT2 phenotype. However, the molecular mechanisms of these correlations were unknown until now. The previously reported interaction of H1 antigen (Fuc-α1,2-Gal-β1,3-GlcNAc) with the most common human rotavirus P genotype P[8] that has been further characterized here at the structural level, highlights the importance of the secretor phenotype on the incidence of rotavirus diarrhea. We have determined the characteristics of this interaction, acknowledging a new binding site for H1 in VP8* common for all the members of P[II] genogroup. Our results show that physical interaction between the H1 antigen and P[8] rotavirus occurs through the precursor side of the molecule (LNB), reinforcing the idea that the main carbohydrate-protein contacts are made via the *N*-acetyl-glucosamine moiety [7]. NMR studies on A-antigen binding of P[9] and P[14] VP8*, demonstrated that the L-fucose moiety does not make contacts with VP8* and rather it remains exposed to the solvent with a high degree of flexibility [21]. However, in the same study VP8* from genotypes P[4] and P[6], that did not recognize A-antigen in our assays, were shown to bind this antigen and L-fucose-protein contacts were evidenced [21]. Structural data from P[4] and P[6] VP8* in complex with LNFPI also showed a limited but direct interaction of the α1,2-linked L-fucose with the protein, namely via the R209 residue, which is conserved in all proteins from genogroup P[II] [12]. Due to the minimal interaction of the secretory L-fucose to VP8*, the authors of this study hypothesize that this glycan moiety has a low contribution to binding affinity and that a strong interaction would be expected for the unfucosylated H1 precursor, explaining the epidemiological studies that do not correlate the FUT2 status to infection by P[4] and P[6] genotypes [22]. Contrarily to this, we show that although the L-fucose moiety of H1 makes indirect contacts with P[8] VP8*, it stabilizes the competent conformation of the LNB moiety to interact with the sugar binding residues, resulting in two-fold lower Kd_a_ for H1 compared to LNB. This small but significative difference may be of relevance in the viral susceptibility context between secretors and non-secretor (FUT2^-/-^) individuals. Furthermore, a weaker interaction to LNB may also explain why infection of P[8] rotaviruses can occur, at a lower level, in non-secretor individuals [23] and it also accounts for the inhibitory effect of LNB in *in vitro* rotavirus infection reported here.

The previously reported interaction of the P[8] genotype with Lewis^b^ identified by ELISA assays [6], could not be reproduced in our experiments. The structural evidence obtained here and in the analyses of the P[4] and P[6] structures in complex with LNFPI [12] argues against interaction with Lewis^b^, which differs with H1 in the presence of an extra L-fucose α1,4-linked to *N*-acetyl-glucosamine that generates steric hindrances to the interaction. These discrepancies, together with the differences found between ELISA and SPR for H1 binding in the different P[8] lineages, suggest that simple qualitative ELISA tests do not always provide reliable results and that other techniques need to be implemented in order to assess VP8* affinities for HBGAs. However, structural data from P[4] and P[6] genotypes predict interaction with A- and B-types HBGAs, as the *N*-acetyl-galactosamine (A-type) and galactose (B-type) located at the non-reducing ends in these glycans do not make any steric hindrance [12]. This coincide with our observation that P[8] VP8* from different lineages interact with blood group A trisaccharide and it is also supported by modelling additional *N*-acetyl-galactosamine in H1 bound to P[8]_c_ VP8* (S6 Fig).

We showed that variations in the binding domain in the P[8] lineages exist that justify the differences in the affinity for H1 and LNB as measured by SPR. Additionally, even if the architecture of the binding site is similar for most P[8] lineages and other genotypes belonging to the P[II] group, other protein residues outside this site may possess epistatic effects over the capacity of the binding pocket to accommodate H1 and LNB, explaining the diverse affinities among P[II] genotypes and in the different P[8] lineages. This is exemplified by the fact that we were able to associate a residue that does not participate in direct protein-ligand contacts (Valine 173) in P[8]_wa_ VP8* to its lower affinity for H1 and LNB and that VP8* from the vaccine strain Rotateq (lineage II), although sharing identical key binding residues to lineage III, do not bind H1 [19]. It is postulated that subtle changes in residues within and outside the defined pocket leads to a fine tuning in HBGA affinities that may ultimately impact host infection capacity. Epidemiologic studies revealed the occurrence of P[8] lineages I, II and III as the major circulating rotavirus with a prevalence of the lineage III [4, 24–26], while lineage IV is rarely found [25]. It is worth mentioning that this lineage, which is phylogenetically distant [4], carries some differences in the H1 binding pocket and showed low affinity to H1, has been isolated from few countries but it seems to be rapidly expanding [27]. Many studies have focused on the rotavirus genotypes circulating before and after the introduction of rotavirus vaccination programs [28, 29], but no work addressed the question of whether a link exists between the secretor status and the incidence of different P[8] lineages. In an study on the effect of the FUT2 status on rotavirus gastroenteritis it was shown that 100% of the patients (n = 51) were secretor positive compared to a healthy control or a group of non-rotavirus gastroenteritis patients (14–19% of non-secretors) and that all rotavirus involved were P[8] from lineage III [30]. Studies on the dynamics of G1P[8] rotavirus in a western population showed that ancient strains were Wa-like (lineage-I) and that new lineages emerged since late nineties [4], although the three main lineages are co-circulating nowadays in most geographical locations. Some authors have argued that the lack of (or low) interaction of Wa-like strains to H1 may help these viruses to prevail, because they do not discriminate by the secretor status [21], but our results suggest that only affinities for the receptor may be varying within the different P[8] lineages and it is not known how this may impact viral fitness. Notably, the amino acids comprising the H1/LNB binding pocket fall outside the defined epitopes in VP8* that elicit protection (S8 Fig). Mutations in VP8* which result in antigenic variants that could escape neutralizing antibodies are frequently isolated [24–26], but they are not affecting the H1/LNB interacting residues defined here. The impact of the different rotavirus P[8] lineages in the population depending on the secretor status deserves further studies in order to ascertain if the prevalence or co-circulation of different P[8] lineages responds to an adaptation to the HBGA profiles of the different hosts.

Despite the need for more exhaustive research on the relevance of HBGA and host specificity/infectivity in P[8] rotavirus, surface glycans possess a clear application in the development of antiviral strategies. It is established that human milk, in addition to other antiviral components, carry a set of oligosaccharides (human milk oligosaccharides; HMO) that share structural similarities to HBGA [31] and could act as anti-adhesins by competition with pathogen ligands at the mucosa. This blocking ability by soluble carbohydrates resembling rotavirus ligands has been evidenced. HMO were shown to inhibit binding of VP8* from P[6] and P[11] genotypes [32]; P[8] and P[4] genotypes infection is inhibited by the HMO 2'-fucosyllactose, 3'-sialyllactose and 6'-sialyllactose [33]; LNFPI inhibited infection of P[19], P[4], P[6] and P[8] genotypes [7], while we showed that LNB inhibited infection of Wa strain *in vitro*. The anti-adhesin potential of this simple disaccharide (LNB) is susceptible for being exploited in antiviral strategies. LNB is present in human milk in its free form [34] but mainly as a building block of type I HMO, which are predominant in human milk over type II HMO (based on LacNAc), which are characteristic of other mammals and primates [31]. Thus, LNB has been considered the human milk ‘bifidus factor’, and many bifidobacterial species from the infant gastrointestinal tract have the enzymatic machinery for its metabolism [35]. LNB would not only act as a bifidobacteria-stimulating prebiotic but also as a viral anti-adhesin to counteract rotavirus infection. Furthermore, its relatively simple synthesis, which can be undertaken enzymatically and by metabolic engineering approaches [36], makes this disaccharide a candidate for the development of new functional foods (e.g. improved infant formula). In this respect, it is important to consider that high affinity constants (in the mM range) have been determined for free oligosaccharides binding to VP8* [12, 21] and, in order to obtain good competitors, conjugated multivalent oligosaccharides seem to be a better option.

Detailed determination of the interactions between viruses and their host is crucial to develop appropriate antiviral strategies. We have defined the molecular interactions of P[8] VP8* from human rotavirus with its ligand HBGA giving a physical explanation as to why the secretor status influences rotaviral infectivity. Notwithstanding, extra structural elements beyond the identified binding site in VP8* are probably responsible for modulating HBGA interactions within P[8] lineages. Dissection of additional VP8* structural features affecting ligand binding is under way.

## Materials and methods

### Expression and purification of rotavirus VP8* proteins

The VP8* (amino acids 64–224 from the VP4 protein of rotavirus) belonging to the P[4], P[6], P[8], P[9], P[11], P[14] and P[25] genotypes were cloned into the, pGEX-2T, expression vector (GE Healthcare) in order to express N-terminal GST fusions.

To amplify P[4], P[6], P[8], P[9] and P[14] VP8*s coding region, RNA was extracted from human stool samples (collected at Hospital Clínico Universitario de Valencia) containing rotavirus of known P genotype using the Trizol reagent following the standard procedure (Invitrogen). Viral RNA was retro-transcribed using the SuperScript Reverse Transcriptase (Invitrogen) and random-primers, and the cDNA was amplified by PCR using Pfu polymerase (Stratagene) with primers detailed in S5 Table. The cDNAs were finally cloned into pGEX-2T (GE healthcare) vector after digestion with BamHI (ThermoFisher). The VP8* genes from genotypes P[4], P[11] and P[25] were purchased as synthetic genes from Gene-ART technologies (ThermoFischer). The expression level of the VP8* protein from the clinical sample P[4] VP8* genotype was very low in *E*. *coli* and its codon usage was optimized. P[11] VP8* and P[25] VP8* were not available as clinical samples. The recombinants GST::VP8* proteins were expressed in *E*. *coli* BL21 (DE3) (Novagen) and purified by affinity chromatography using GSTrap columns coupled to an ÄKTA prime FPLC system (GE Healthcare). All sequences are included as fasta files in the supplementary data.

### Site directed mutagenesis

Selected residues in the GST::VP8* P[8] were replaced for alanine or valine according to the structural data of the LNB binding site. Four mutants (M1–M4) were constructed using a Quick-Change site-directed mutagenesis kit (Stratagene) and appropriate oligonucleotides (S5 Table), and the DNA changes were confirmed by DNA sequencing. Mutant M1, M2 and M3 contained changes in the codons for tryptophan 174, arginine 209 and glutamic 212 residues, respectively, that introduced an alanine at each position. Mutant M4 substituted isoleucine 173 by valine.

### VP8 glycan binding assays

A panel of biotinylated sugar antigens including Le^a^, Le^b^, Le^c^ (lacto-*N*-biose; LNB), H type-1, H type-2 and blood group A and B trisaccharides were purchased from Glyconz (Fig 1 and S1 Table). These glycans are biotinylated neoglyconjugates of a poly[N-(2-hydroxyethyl)acrylamide] (PAA) with a size from 30 to 50 KDa. This forms a flexible polymer ideal for a multivalent presentation of glycans. Immobilized streptavidin F96 black plates (Nunc) were coated with the biotinylated oligosaccharides (2 μg/ml) in milli-Q water and incubated during 1 hour at 37°C. After functionalization the plates were washed once with PBS containing 0.05% of Tween 20 (PBS-T) and the VP8* proteins were added (10 μg/ml) and incubated at 4°C overnight. After three washes with PBS-T, a rabbit polyclonal antibody anti GST (1:1,000) (Abcam) was added and the plates were incubated one hour at 37°C. Then, the plates were washed three times with PBS-T and incubated for 1 h at 37°C with 1:10,000 dilution of horseradish peroxidase (HRP)-conjugated goat anti-rabbit (Abcam). After three washes with PBS-T, the binding was detected using QuantaBlue reagent (ThermoFisher) kit, as recommended by the manufacturer. Fluorescence units were registered by a MultiScan microplate reader. All the binding assays were performed in triplicate.

The EC_50_ binding of the VP8* from the clinical (P[8]_c_) and Wa (P[8]_Wa_) genotypes to the H type-1 and to its precursor (LNB, Le^c^) was determined incubating two fold serial dilutions of the VP8* proteins, ranging from 100 μg/ml to 1.5 μg/ml. The binding assays were performed in triplicate using the protocol described above.

### Binding blocking assays

To confirm the binding of the VP8* from the P[8] genotype to the H type-1 precursor LNB, a blocking assay was performed using soluble LNB and its related disaccharide galacto-*N*-biose (Gal-β-1,3-GalNAc; GNB) produced and purified in our laboratory as previously described [36].

Streptavidin microtiter plates were coated with biotinylated H type-1 antigen or LNB at 2 μg/ml with water and incubated for 1 hour at 37°C, followed by an overnight incubation at 4°C. Blocking assays were performed in parallel, using glass tubes containing the P[8]c and P[8]_Wa_ VP8* protein at their EC_50_ for each ligand and 20 mM of each of the soluble disaccharides (LNB and GNB) and monosaccharides (D-galactose, GlcNAc and GalNAc). A positive binding control without sugar was also included. The tubes containing the mixes of VP8* with sugars were maintained 1 hour at 37°C, followed by an overnight incubation at 4°C. The next day the coated streptavidin plates were washed with PBS-T, the VP8*-sugar solutions were added to the plates and incubated during 4 hours at 4°C. The plates were washed three times with PBS-T and detection of the interactions was performed as described above. The results are presented as the percentage (%) of binding of each condition compared to the binding of the positive binding control (without blocking sugar). All experiments were performed in triplicate.

### Production of rotavirus particles

African green monkey kidney epithelial cells (MA104 cell line; ATTC #CRL-2378.1) were used for the propagation of rotavirus Wa strain that belongs to the globally dominant human genotype G1P[8]. Briefly, ten MA104 cells confluent 150-cm^2^ flasks (approximately 1.5 ×10^7^ cells/flask) were infected with Wa strain at a multiplicity of infection (MOI) of ≤ 0.1 and processed as previously described [37]. One hundred ml of medium with 1.5x10^8^ virus/ml were obtained and the viral particles were concentrated by pelleting at 160,000 × g for 1 h at 4°C in a SW 41 rotor (Beckman). The viral pellet was resuspended in TNC buffer (20 mM Tris-HCl pH 8.0, 100 mM NaCl, 1 mM CaCl_2_) for triple-layered particles (TLP) or in TNE (20 mM Tris-HCl, pH 8.0, 100 mM NaCl, 1 mM EDTA) for double-layered particles (DLP).

### Rotavirus triple-layered (TLP) and double-layered particles (DLP) glycan binding assays

An ELISA-like binding assay was employed to determine the binding ability of rotavirus TLP and DLP to the H type-1 antigen and to its precursor LNB. Streptavidin plates were coated with the biotinylated oligosaccharides as described above. After washing with PBS-T, two fold serial dilutions of TLPs and DLP were added to the plate (ranging from 10 μg/ml to 0.078 μg/ml). The TLP were always maintained in TNC-T (20 mM Tris, 100 mM NaCl, 1 mM CaCl_2_, 0.05% Tween 20, pH 7,4) buffer and the binding and washing steps were always carried out in this solution. DLPs assays were carried out in Tris-buffered saline buffer with Tween 20 (TBS-T, 20 mM Tris, 100 mM NaCl, 0.05% Tween 20, pH 7.4). TLP and DLP were incubated in the plate overnight at 4°C. After binding, the plates were washed three times in TNC-T (for TLP) or TBS-T (for DLP) with 0.05% Tween 20 (TNC-T and TBS-T), and a mouse anti-VP6 antibody was added at 1:100 in TNC-T or TBS-T and incubated 1 h at 37°C. The plates were then washed three times with TNC-T or TBS-T, and a HRP-conjugated anti-mouse IgG was added at 1:10.000 and incubated at 37°C for 1 h. After three final washes the binding was revealed by QuantaBlue reagent (ThermoFisher) following the manufacturer recommendations. Fluorescence units were recorded by a MultiScan microplate reader.

### Blocking of rotavirus infection in MA104 cells

The G1P[8] Wa strain was tested on MA104 cells. The sugars LNB, GNB, GlcNAc, GalNAc, D-galactose and L-fucose were tested for their effect on rotavirus infectivity. The oligosaccharides were previously heat sterilized at 99°C for 10 min and then dissolved in serum-free DMEM containing 1 μg/ml trypsin. Serum-free DMEM containing 1 μg/ml without oligosaccharide was used as a control in each experiment.

The effect of different mono- and disaccharides on rotavirus infectivity was assessed through standard fluorescent focus assays on MA104 cells [37]. The dilution of Wa virus stocks that yielded ~150 focus-forming units/well was first established. Then, sugars were added during virus inoculation at a final concentration of 5 mg/ml, incubated for 1 hour, and unbound virus was removed by washing with FBS-DMEM. The cells were allowed to be infected for 16h, washed once with PBS and fixed with 100% methanol. A mouse anti-VP6 primary antibody (1:50 dilution in PBS containing 3% BSA) was added and incubation proceeded for 30 min at room temperature with gentle rotation. A secondary antibody anti-mouse IgG-FITC (Sigma F4143) diluted 1:128 in PBS containing 3% BSA was added and incubated for 30 min at room temperature with gentle rotation. Individual fluorescence foci were counted on an inverted fluorescence microscope with a FITC-compatible filter. Infectivity in the absence of oligosaccharides served as the control. Each experimental condition was tested a minimum of 2 times, with technical triplicates for each oligosaccharide. The means and SD from a minimum of 6 determinations are represented for each condition. Virus titer measured in the absence of oligosaccharides was considered to be 100% infectivity, and changes in virus titer in the presence of sugars were expressed as percentage of infectivity compared with no sugar treatment.

### Surface plasmon resonance (SPR) analysis

The affinity assays were based on SPR and performed in a Biacore T100 instrument (GE Healthcare). H1 PAA-biotin and LNB PAA-biotin were diluted to a concentration of 1 mg/ml in water and captured with streptavidin present in a SA sensor chip (GE Healthcare). H1 was immobilized in channel 2 (630 RU) of the sensor chip and LNB was immobilized in channel 4 (624 RU). The channels 1 and 3 were used as the reference surfaces for channels 2 and 4, respectively. The immobilization process was performed by conditioning the sensor chip surface with three consecutive 1-minute injections of 1 M NaCl 50 mM NaOH before biotinylated ligands were immobilized at a flow rate of 15 μl/min. The affinity assays of VP8* polypeptides to biotinylated sugars were performed at 10°C using 1X HBS-EP^+^ buffer (0.01 M HEPES pH 7.4, 0.15 M NaCl, 3 mM EDTA, 0.005% Surfactant P20), a flow rate of 5 μl/min with 2700 seconds of contact time and a dissociation time of 1800 seconds. The regeneration step consisted in a wash step with 10 mM Glycine-HCl pH 2 for 20 seconds at the same flow rate. The assays were performed with purified VP8* at different concentrations (45; 137; 411; 1,234; 3,703; 11,111; 33,333; 100,000 and 200,000 nM). Each run included three blanks without sample. The affinity data were obtained after analysis of sensorgrams performed with the BIAevaluation 2.0 software (GE Healthcare). Since multivalent oligosaccharides are immobilized on a sensor chip surface, avidity and rebinding effects can take place and apparent affinity constants (Kd_a_) are calculated with this experimental setup. Kd_a_ values were obtained from the steady-state kinetics experiment as the ligand concentration needed to achieve a half-maximum binding at equilibrium. The experiments were made in triplicate. Graphical representation of signal/concentration curves were plotted using GraphPad Prism 6 for MacOsX.

### Crystallization and data collection

The crystals were grown as hanging drops at 21°C with a vapour-diffusion approach. Initial crystallization trials were set up in the crystallogenesis service of the IBV-CSIC using commercial screens JBS I, II (JENA Biosciences) and JCSG+ (Molecular Dimensions) in 96-well plates. Crystallization drops were generated by mixing equal volumes (0.3 μl) of P[8]_c_ VP8* protein solution and the corresponding reservoir solution, and were equilibrated against 100 lμl reservoir solution. Both P[8]_c_ VP8* Apo structures were crystallized at 10 mg/ml. VP8* Apo1 was crystallized in a reservoir solution of 1.2 M (NH_4_)_2_SO_4_, 3% iso-propanol and 0.1 sodium citrate pH 4.6, whereas VP8* Apo2 was crystallized in 1.5 M Li_2_SO_4_ and 0.1 M Tris-HCl pH 6.5. In both cases 2 M Li_2_SO_4_ was used to cryoprotect the crystal when freezing in liquid nitrogen. For the crystallization in presence of glycans, the ligands were mixed with the protein at 10 mM final concentration of ligand and 10 mg/ml of protein final concentration. P[8]_c_ VP8* LNB was crystallized in a reservoir solution consisting in 25% PEG 3,350 0.1 M Bis-Tris pH 5.5. The cryosolution used for crystal freezing was its reservoir solution increased up to 35% PEG 3,350. P[8]_c_ VP8* H1 was crystallized against a a reservoir solution consisting in 25% PEG 6,000, 0.1 M Na-HEPES pH 7.5, 0.1 M LiCl, and PEG 6,000 was increased up to 35% for cryoprotection. X-ray diffraction was carried out at 100K at Alba (Cerdanyola, Barcelona, Spain) and DLS (Didcot, UK) synchrotrons and the best data sets used to solve the structures were collected at the indicated beamlines and wavelengths (Table 2). Diffraction data was processed and reduced with Mosflm[38] and Aimless[39] programs from the CCP4 suite [40]. The data-collection statistics for the best data sets used in structure determination are shown in Table 2.

### Model building

P[8]_C_ VP8* Apo1 structure was solved by molecular replacement carried out with the program Phaser [41] and using the structure of VP8* from CRW-8 porcine rotavirus (PDB 2I2S[20]) as a model. Initial phases from the molecular replacement were used to manually build the P[8]c VP8* structure with Coot [42]. P[8]_c_ VP8* Apo1 structure was then used as a model for molecular replacement to solve the P[8]_c_ VP8* Apo2, P[8]c VP8*LNB and P[8]_c_ VP8*H1 structures. All the final models were generated by iterative cycles of refinement using the Refmac [43] and manually optimization with Coot. Data refinement statistics are given in Table 2. The crystals exhibited good quality control parameters and excellent stereochemistry. Atomic coordinates and structure factors have been deposited in the Protein Data Bank (PDB) with ID numbers 6H9W, 6H9Z, 6H9Y and 6HA0 for P[8]_c_ VP8* Apo1, P[8]_c_ VP8* Apo2, P[8]_c_ VP8*LNB and P[8]_c_ VP8*H1, respectively. Structure Superposition and RMSD calculations were carried out with Superpose [44] from CCP4 suite.

### Statistical analysis

To assess statistical differences in the ELISA-like binding experiments where many groups were compared an ANOVA test was performed. To analyze significative differences in the Kd_a_ values obtained by SPR an unpaired t-test was applied. All statistical analyses were performed with GraphPad Prism version 6.0 for MacOsx (GraphPad Software). *p* values <0.05 were considered to be statistically significant.

### Ethics statement

This study was conducted with the approval of the Ethics Committee of the University of Valencia (code H1544010468380).

The human stool samples from Hospital Clínico Universitario de Valencia were anonymized previously to their inclusion in the present study.

## Supporting information

S1 Fig Coomassie blue stained 12% SDS-PAGE gel showing the different GST::VP8* proteins used in the present study. The genotype of each one of the proteins is indicated as well as the molecular weight marker (Mw). The molecular weights (in kDa) of the marker are indicated at the right of the gel. The P[8]_Wa_ corresponds to the lineage I and the P[8]_c_ corresponds to the lineage III strain RVA/Human-wt/VLC/3455/2015/[G1P8]. Lineage IV correspond to strain RVA/Human-tc/BGD/MMC71/2005/G1P[8].
(PPTX)

S2 Fig ELISA-like binding assays of rotavirus VP8* from different genotypes to a panel of biotinylated sugars. The interaction pairs are indicated in each panel from **panel a** to **panel j**.
(PPTX)

S3 Fig Characterization of the interaction of P[8]_c_ and P[8]_wa_ VP8*s to the H1 antigen and LNB by ELISA. The graph shows the concentration-dependent binding of VP8* from the clinical isolate (P[8]_c_) and from the cultivable Wa strain (P[8]_Wa_) to the H1 antigen and to its precursor lacto-*N*-biose (LNB).
(PPTX)

S4 Fig P[8]_c_ VP8* has a preformed sugar binding pocket coinciding with the observed in viruses of the same genogroup but differing from the observed in other genogroups. **a** The backbone structures of P[8]_c_ VP8* in its apo form (Apo1 and Apo2 in orange and magenta, respectively), and in complex H1 (green) and LNB (blue) are superimposed. The glycans bound to P[8]_c_ VP8* are shown in stick representation with carbon atoms colored according to the corresponding structure. **b** Superposition of the backbone structures of P[8]_c_ VP8* H1 (green) with VP8* apo forms of the linage I P[8]_Wa_ (magenta; PDB 2DWR[20]) and P[8]_Rotarix_ (blue; PDB 5JDB[19]). H1 glycan is shown in sticks with carbon atoms colored in green. **c** Superimposition of VP8* structures form different members of PII genogroup. The backbones structures of VP8* proteins in its apo form from P[8]_Wa_ (magenta; PDN 2DWR[20]) and P[8]_Rotarix_ (blue; PDB 5JDB[19]) or in complex with different glycans from P[8]_c_ (green), P[19] (orange; PDB 5VKS[7]), P[6] (cyan; PDB 5VX9[12]) and P[4] (gray; PDB 5VX5[12]) are superimposed and the bound glycans (H1 or LNFPI) are shown in stick representation with carbon atoms colored according to the corresponding structure. **d** Superimposition of backbone structures of P[8]_c_ VP8* (green), P[14] VP8* (pink; PDB 4DS0[9]) and P[11] VP8* (light blue; PDB 4YG0[16]) bound to H1, A-type and LNnT glycans (in sticks), respectively, shown that sugar biding pocket localization differ between genogroups.
(PPTX)

S5 Fig Characterization of the interaction of M1, M2, M3 and M4 VP8*mutant variants with the H1 antigen and lacto-*N*-biose (LNB) by SPR. **a** to **f** Lack of binding of mutants M1 to M3 to the H1 antigen (**a**, **c** and **e**) and to LNB (**b**, **d** and **f**), confirming the functionality of the VP8* binding pocket. **g** and **h** show binding and obtained Kd_a_ of the M4 mutant (Ile175Val P[8]_c_) to H1 and LNB, respectively.
(PPTX)

S6 Fig Structural evidences of P[8]_c_ VP8* sugar selectivity. Modeling of the Lewis^b^ (**a**) and A-type (**b**) antigens in P[8]c VP8* H1 structures shows that the Lewis^b^ Fuc (in sticks with carbon atoms colored in cyan) is projected from the H1 glycan (in sticks with carbon atoms in green) towards the VP8* protein (in stale blue carton highlighting the protein surface in white semitransparent representation) clashing with different sugar recognizing residues as T185 and E212. In the case of the A-type I antigen, the presence of an additional GalNAc moiety (in sticks with carbon atoms colored in magenta) has not steric problems since it is project towards the solvent.
(PPTX)

S7 Fig Minimal movements induced by sugar binding to P[8] VP8*. The structures of P[8]_Wa_ (pink; PDN 2DWR[20]), P[8]_Rotarix_ (light blue; PDB 5JDB[19]) and P[8]_c_ (orange) in its apo forms are superimposed with P[8]_C_ in complex with H1 (green) and the backbone of the structural elements conforming the glycan binding site are shown. The bound H1 antigen is represented in sticks with carbon atoms in yellow (GlcNAc), green (Gal) and slate blue (Fuc). The residues interacting with the sugars are shown in stick representation, with carbon atoms colored according to the structural element to which they correspond. Oxygen and nitrogen atoms are colored in red and dark-blue, respectively, in all the structure. A subtle displacement of the loop connecting β9-β10 strands between the glycan free and glycan-bound forms is observed and indicated by and around. Similar displacement is observed in the ligang-binding residue Y169. The loop displacement is facilitated by the presence of two Gly (G170 and G171) residues represented as spheres. The hydrophobic residue (Val or Ile) in the backside of the glycan binding pocket at position 173 is shown as stick.
(PPTX)

S8 Fig Sugar recognizing residues are not antigenic. Two surface representations of P[8]_c_ VP8* rotated 180° are shown highlighting the position of the antigenic residues in Rotarix and RotaTec strains in red and the sugar binding residues in cyan. The H1 sugar is represented in sticks with carbon atoms in yellow (GlcNAc), green (Gal) and slate blue (Fuc).
(PPTX)

S1 Table Biotinylated oligosaccharides utilized in the present study. All biotinylated oligosaccharides were purchased from Glyconz.
(PPTX)

S2 Table RMSD values for the superposition of all the Cα atoms of VP8* structures from different P[II] genogroup rotavirus (Apo or glycan bound). (PPTX)

S3 Table Contacts between the precursor LNB and H1 trisaccharide ligands with the VP8* protein from P[8]c. (PPTX)

S4 Table Comparison of the amino acid positions involved in H1 and LNB binding in different P[8] lineages of VP8*. Residues critical for H1 and LNB interaction were determined in P[8]_c_ VP8* (lineage III). The residues that were mutated to alanine (174, 209 and 212) are shown in bold. The underlined residue (position 173) does not make ligand contacts but differs between lineage I and lineages II, III and IV.
(PPTX)

S5 Table Primer sequences utilized in the present work. *BamHI* restriction site is underlined.
(PPTX)

S6 Table Sequences of the different VP8* in fasta format. (PPTX)
