# Supplementary figures and images for: Unraveling the role of the secretor antigen in human rotavirus attachment to histo-blood group antigens
Source: PLoS Pathog. 2019 Jun 21;15(6):e1007865. doi: 10.1371/journal.ppat.1007865 (PMC6609034; doi:10.1371/journal.ppat.1007865)

## Slide 1
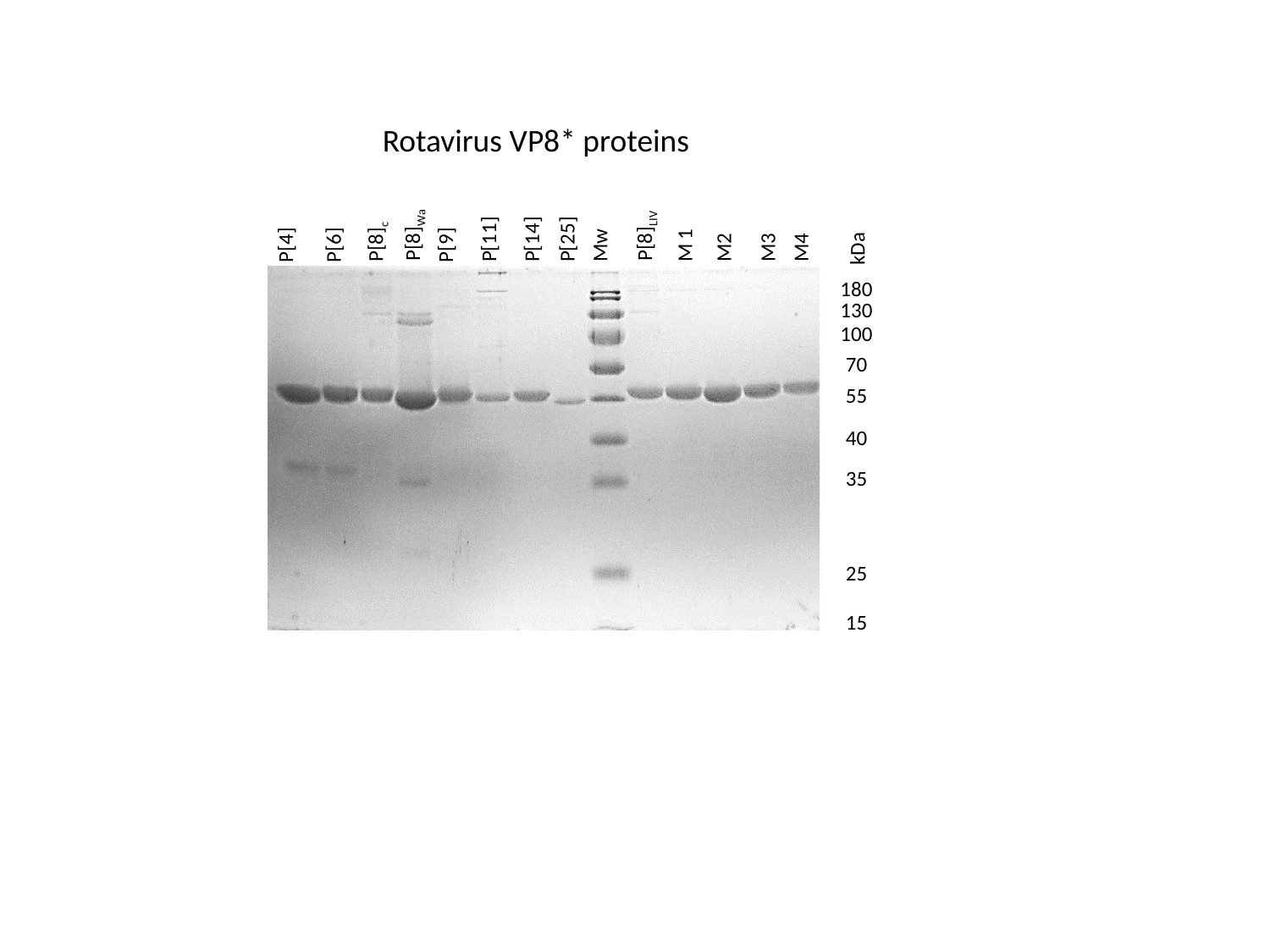

Rotavirus VP8* proteins
P[8]Wa
P[8]LIV
P[11]
P[14]
P[25]
P[8]c
P[4]
P[6]
P[9]
M 1
Mw
M2
M3
M4
kDa
180
130
100
70
55
40
35
25
15

Supplement: S1 Fig — The genotype of each one of the proteins is indicated as well as the molecular weight marker (Mw). The molecular weights (in kDa) of the marker are indicated at the right of the gel. The P[8]Wa corresponds to the lineage I and the P[8]c corresponds to the lineage III strain RVA/Human-wt/VLC/3455/2015/[G1P8]. Lineage IV correspond to strain RVA/Human-tc/BGD/MMC71/2005/G1P[8]. (PPTX) [file ppat.1007865.s001.pptx]

## Slide 1
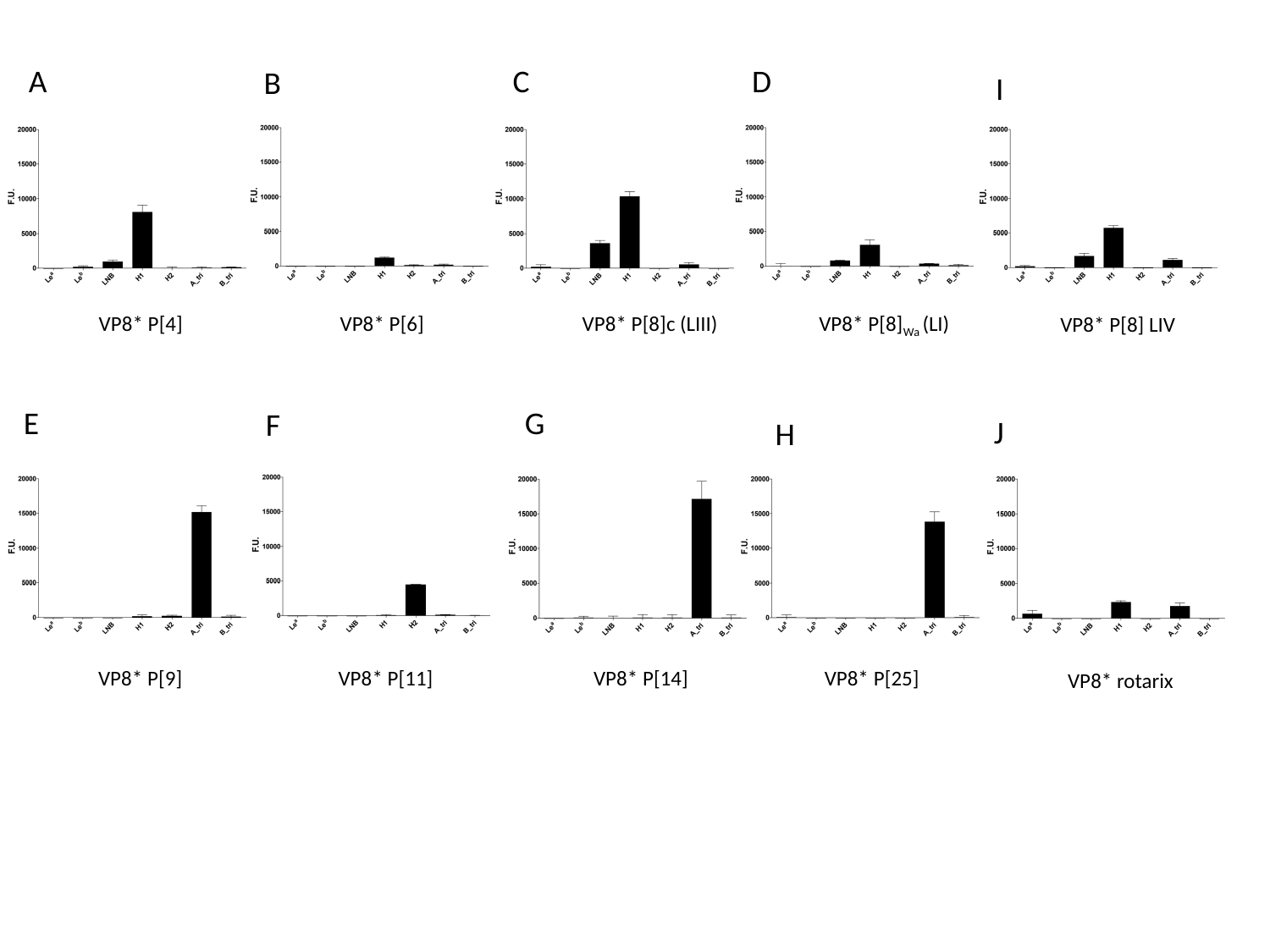

A
C
D
B
I
VP8* P[4]
VP8* P[6]
VP8* P[8]c (LIII)
VP8* P[8]Wa (LI)
VP8* P[8] LIV
E
G
F
J
H
VP8* P[25]
VP8* P[14]
VP8* P[9]
VP8* P[11]
VP8* rotarix

Supplement: S2 Fig — The interaction pairs are indicated in each panel from panel a to panel j. (PPTX) [file ppat.1007865.s002.pptx]

## Slide 1
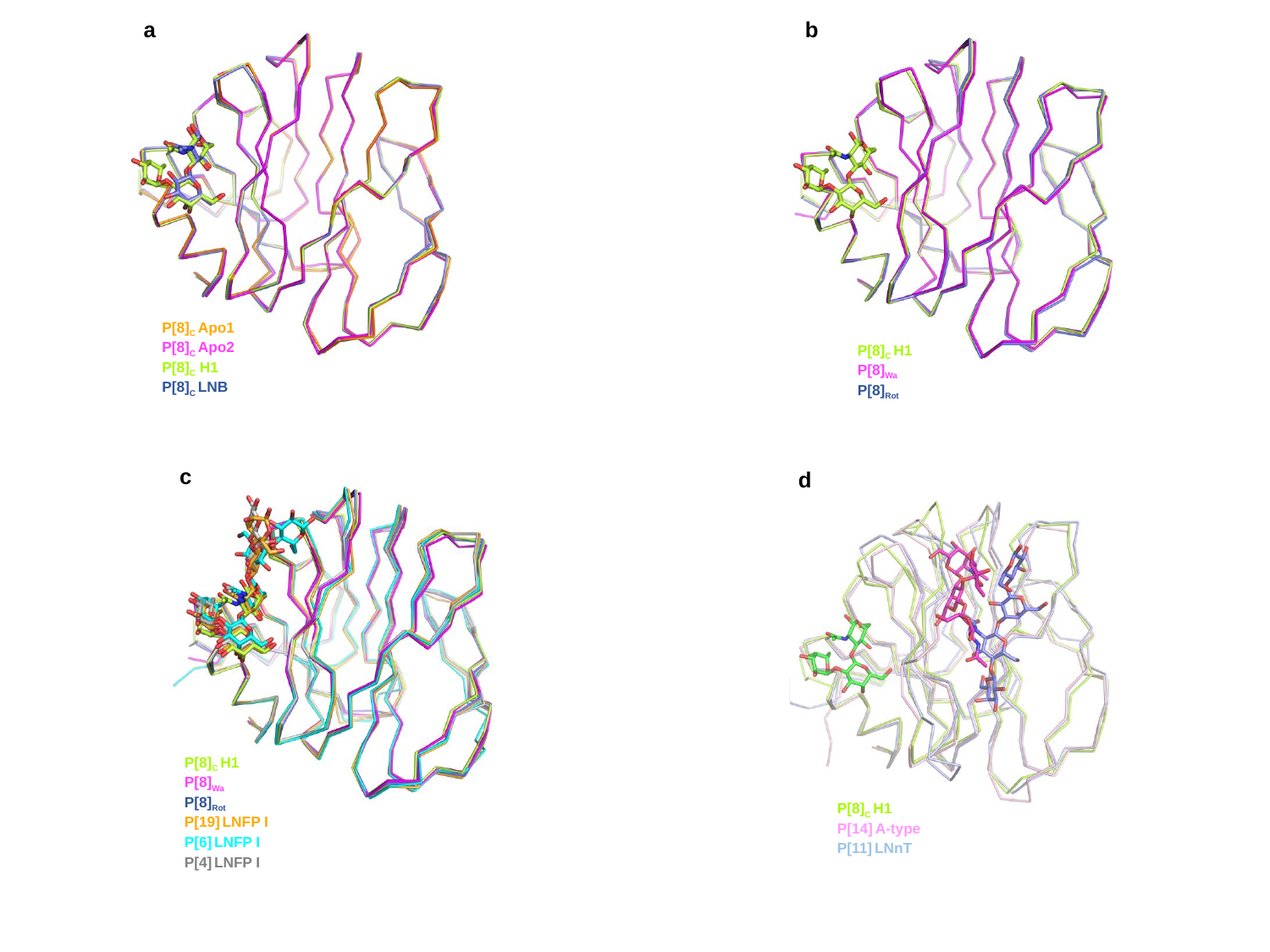

a
b
P[8]C Apo1
P[8]C Apo2
P[8]C H1
P[8]C LNB
P[8]C H1
P[8]Wa
P[8]Rot
c
d
P[8]C H1
P[8]Wa
P[8]Rot
P[19] LNFP I
P[6] LNFP I
P[4] LNFP I
P[8]C H1
P[14] A-type
P[11] LNnT

Supplement: S4 Fig — a The backbone structures of P[8]c VP8* in its apo form (Apo1 and Apo2 in orange and magenta, respectively), and in complex H1 (green) and LNB (blue) are superimposed. The glycans bound to P[8]c VP8* are shown in stick representation with carbon atoms colored according to the corresponding structure. b Superposition of the backbone structures of P[8]c VP8* H1 (green) with VP8* apo forms of the linage I P[8]Wa (magenta; PDB 2DWR[20]) and P[8]Rotarix (blue; PDB 5JDB[19]). H1 glycan is shown in sticks with carbon atoms colored in green. c Superimposition of VP8* structures form different members of PII genogroup. The backbones structures of VP8* proteins in its apo form from P[8]Wa (magenta; PDN 2DWR[20]) and P[8]Rotarix (blue; PDB 5JDB[19]) or in complex with different glycans from P[8]c (green), P[19] (orange; PDB 5VKS[7]), P[6] (cyan; PDB 5VX9[12]) and P[4] (gray; PDB 5VX5[12]) are superimposed and the bound glycans (H1 or LNFPI) are shown in stick representation with carbon atoms colored according to the corresponding structure. d Superimposition of backbone structures of P[8]c VP8* (green), P[14] VP8* (pink; PDB 4DS0[9]) and P[11] VP8* (light blue; PDB 4YG0[16]) bound to H1, A-type and LNnT glycans (in sticks), respectively, shown that sugar biding pocket localization differ between genogroups. (PPTX) [file ppat.1007865.s004.pptx]

## Slide 1
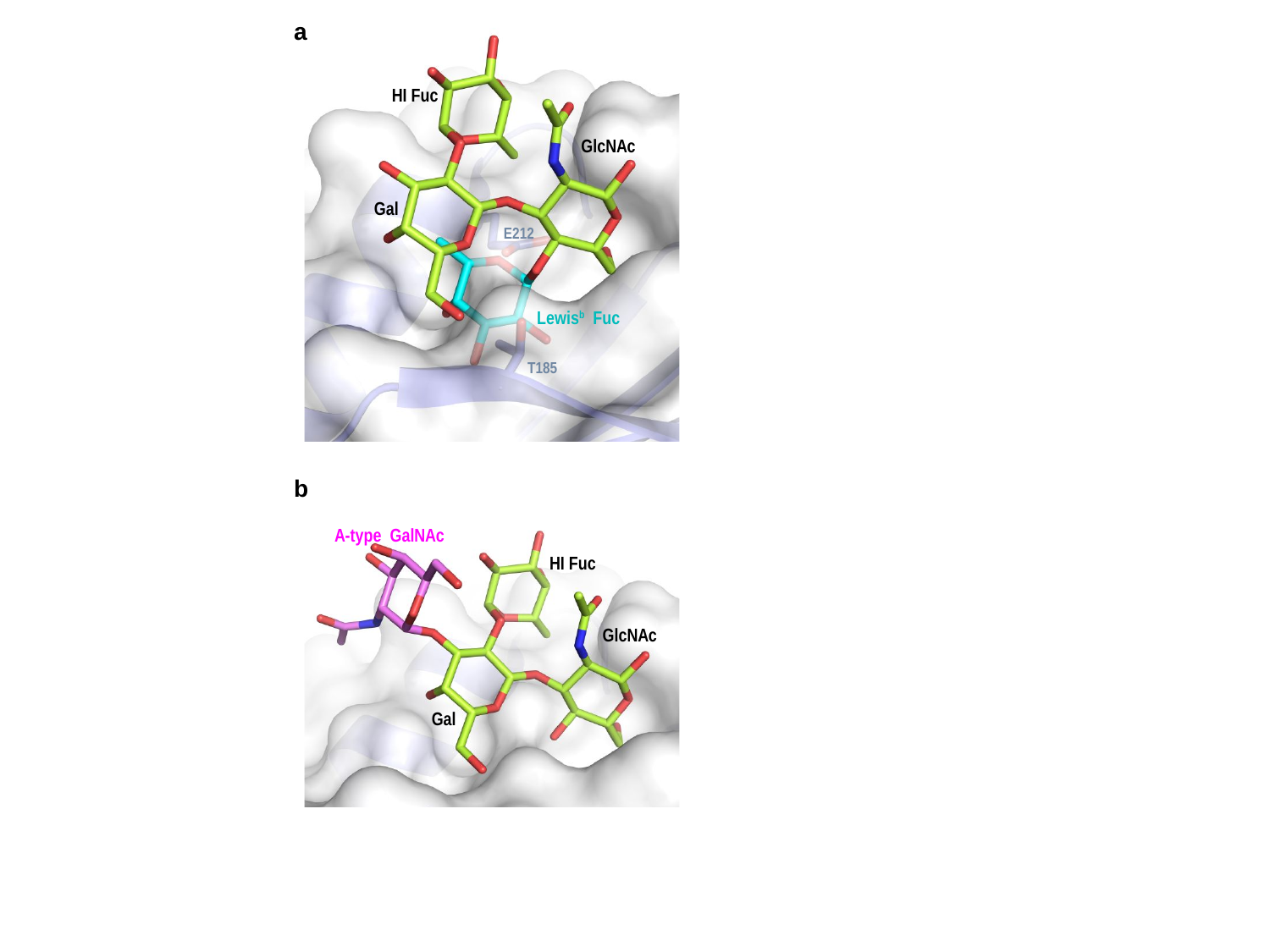

a
HI Fuc
GlcNAc
Gal
E212
Lewisb Fuc
T185
b
A-type GalNAc
HI Fuc
GlcNAc
Gal

Supplement: S6 Fig — Modeling of the Lewisb (a) and A-type (b) antigens in P[8]c VP8* H1 structures shows that the Lewisb Fuc (in sticks with carbon atoms colored in cyan) is projected from the H1 glycan (in sticks with carbon atoms in green) towards the VP8* protein (in stale blue carton highlighting the protein surface in white semitransparent representation) clashing with different sugar recognizing residues as T185 and E212. In the case of the A-type I antigen, the presence of an additional GalNAc moiety (in sticks with carbon atoms colored in magenta) has not steric problems since it is project towards the solvent. (PPTX) [file ppat.1007865.s006.pptx]

## Slide 1
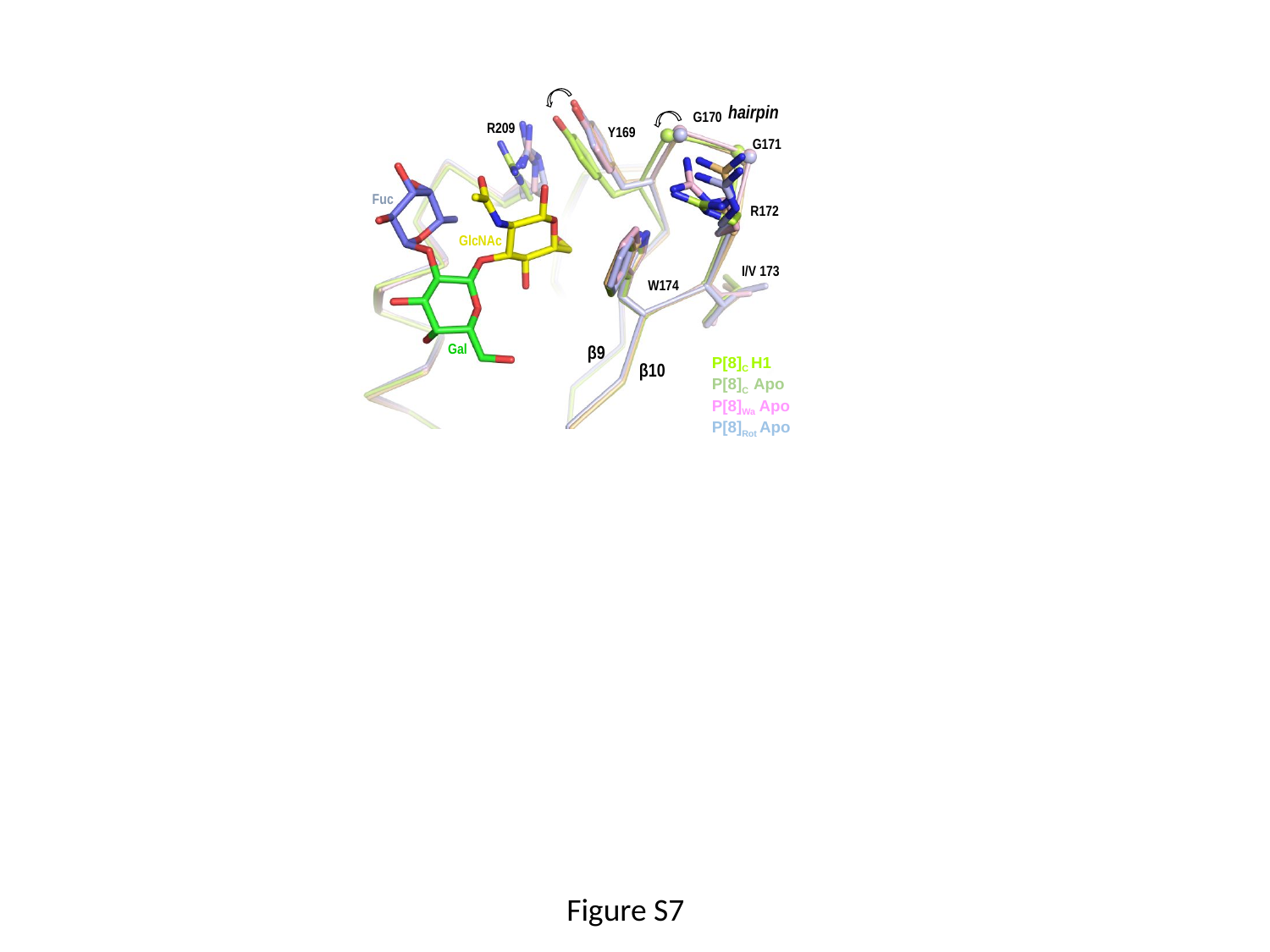

hairpin
G170
R209
Y169
G171
Fuc
R172
GlcNAc
I/V 173
W174
Gal
β9
β10
P[8]C H1
P[8]C Apo
P[8]Wa Apo
P[8]Rot Apo
Figure S7

Supplement: S7 Fig — The structures of P[8]Wa (pink; PDN 2DWR[20]), P[8]Rotarix (light blue; PDB 5JDB[19]) and P[8]c (orange) in its apo forms are superimposed with P[8]C in complex with H1 (green) and the backbone of the structural elements conforming the glycan binding site are shown. The bound H1 antigen is represented in sticks with carbon atoms in yellow (GlcNAc), green (Gal) and slate blue (Fuc). The residues interacting with the sugars are shown in stick representation, with carbon atoms colored according to the structural element to which they correspond. Oxygen and nitrogen atoms are colored in red and dark-blue, respectively, in all the structure. A subtle displacement of the loop connecting β9-β10 strands between the glycan free and glycan-bound forms is observed and indicated by and around. Similar displacement is observed in the ligang-binding residue Y169. The loop displacement is facilitated by the presence of two Gly (G170 and G171) residues represented as spheres. The hydrophobic residue (Val or Ile) in the backside of the glycan binding pocket at position 173 is shown as stick. (PPTX) [file ppat.1007865.s007.pptx]

## Slide 1
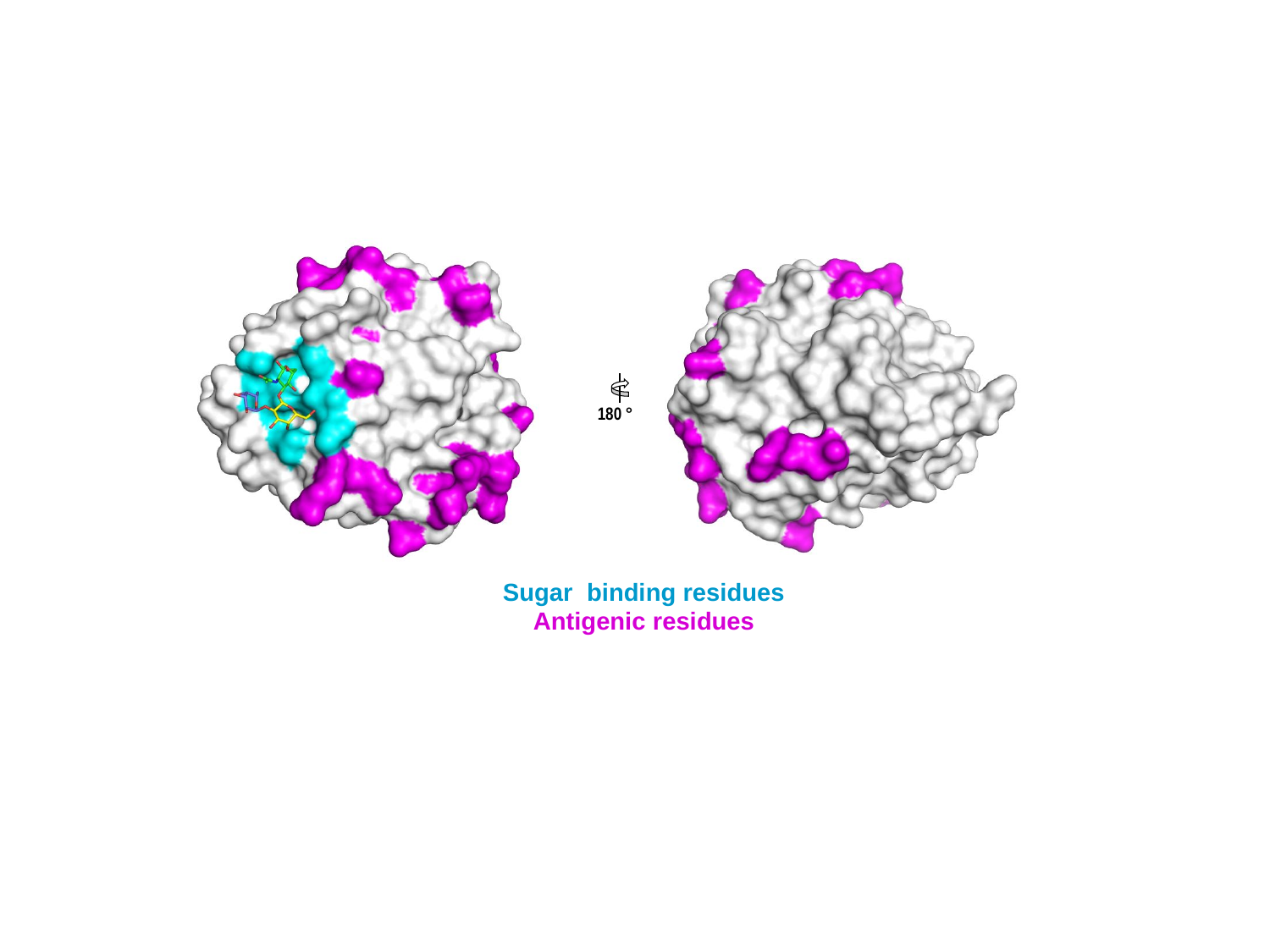

180 °
Sugar binding residues
Antigenic residues

Supplement: S8 Fig — Two surface representations of P[8]c VP8* rotated 180° are shown highlighting the position of the antigenic residues in Rotarix and RotaTec strains in red and the sugar binding residues in cyan. The H1 sugar is represented in sticks with carbon atoms in yellow (GlcNAc), green (Gal) and slate blue (Fuc). (PPTX) [file ppat.1007865.s008.pptx]

## Slide 1
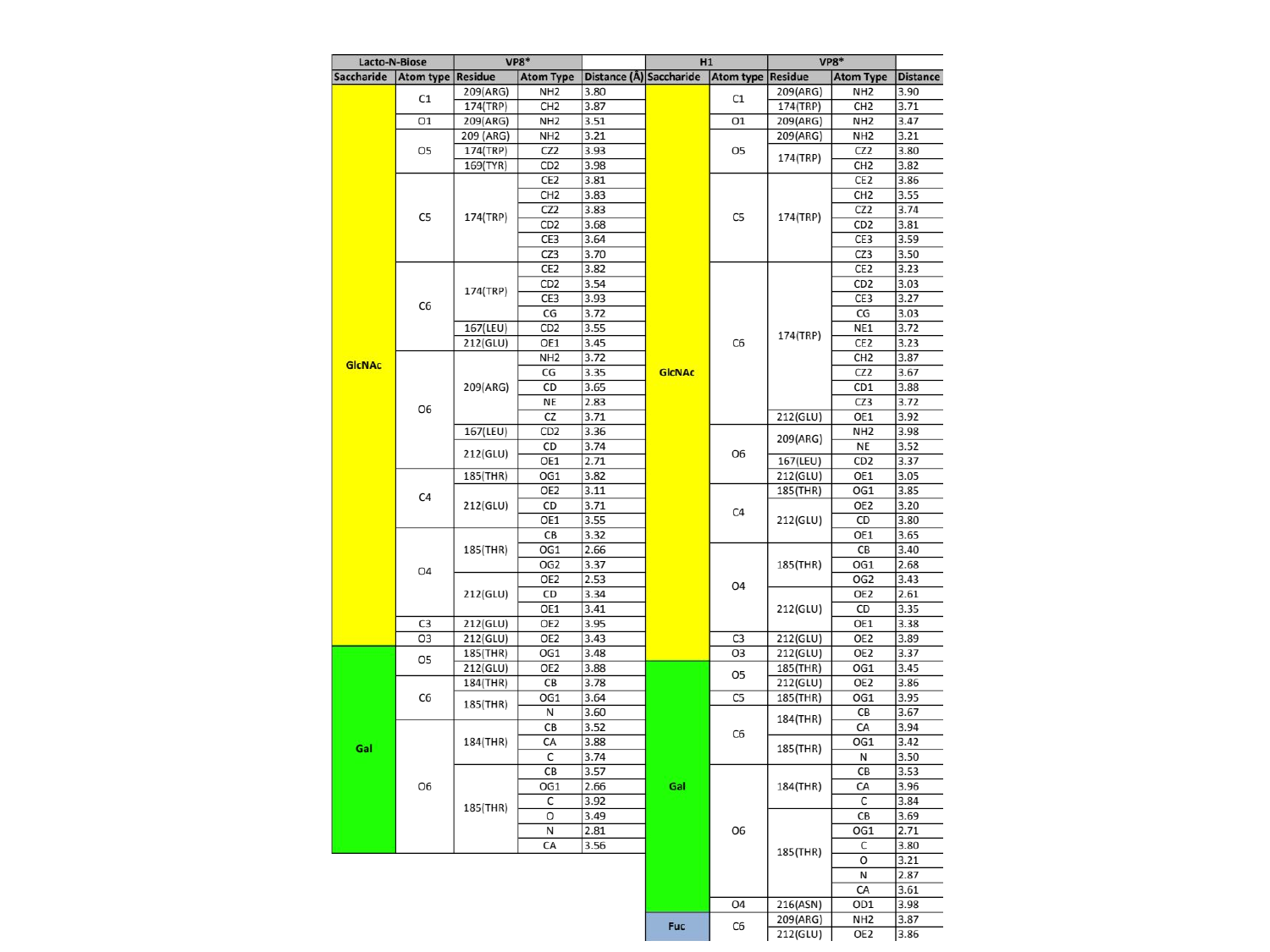

Supplement: S3 Table — (PPTX) [file ppat.1007865.s011.pptx]

## Slide 1
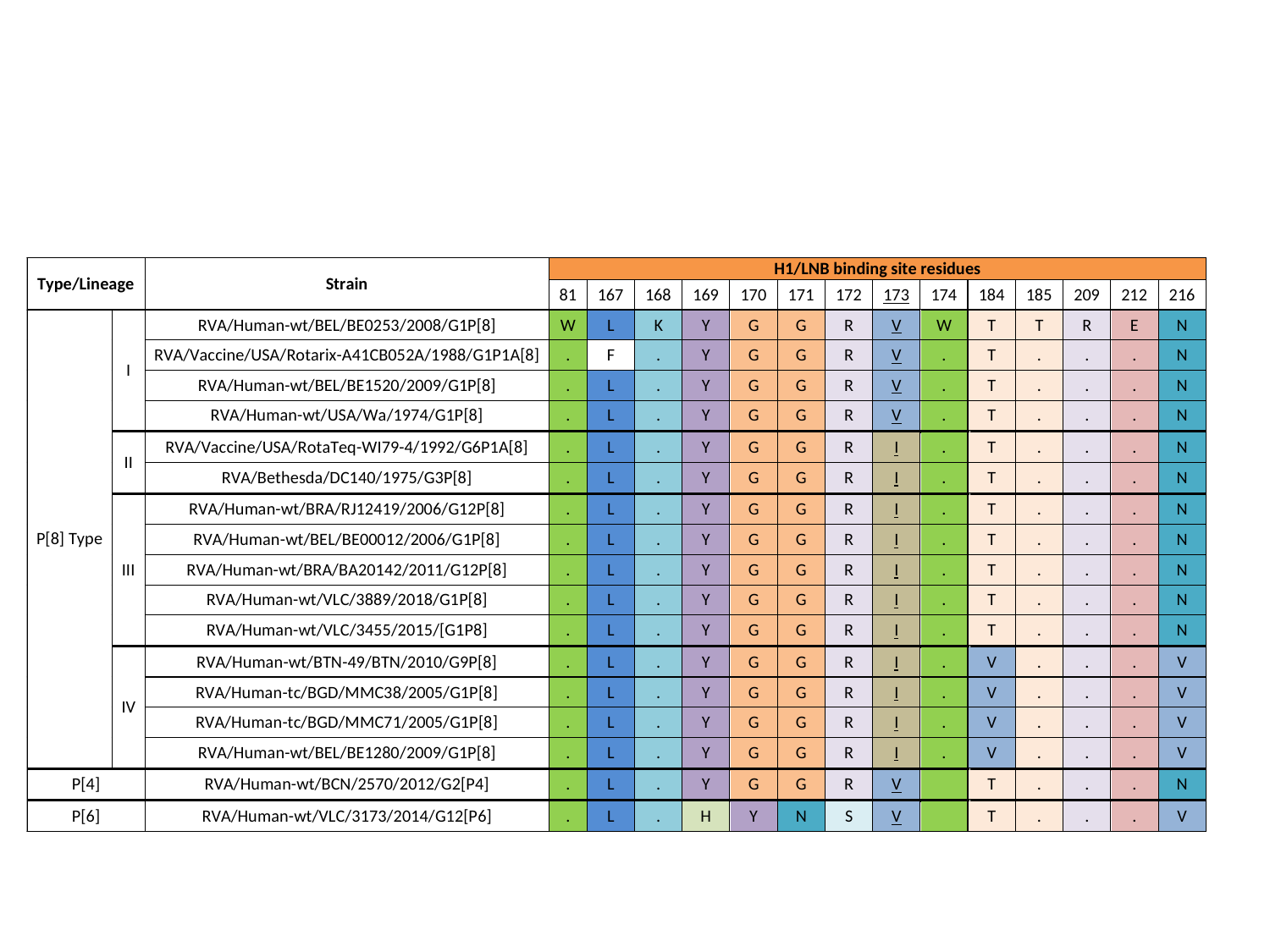

Supplement: S4 Table — Residues critical for H1 and LNB interaction were determined in P[8]c VP8* (lineage III). The residues that were mutated to alanine (174, 209 and 212) are shown in bold. The underlined residue (position 173) does not make ligand contacts but differs between lineage I and lineages II, III and IV. (PPTX) [file ppat.1007865.s012.pptx]
