# Supplementary material for: Unraveling the role of the secretor antigen in human rotavirus attachment to histo-blood group antigens
Source: PLoS Pathog. 2019 Jun 21;15(6):e1007865. doi: 10.1371/journal.ppat.1007865 (PMC6609034; doi:10.1371/journal.ppat.1007865)
Supplement: S3 Fig — The graph shows the concentration-dependent binding of VP8* from the clinical isolate (P[8]c) and from the cultivable Wa strain (P[8]Wa) to the H1 antigen and to its precursor lacto-N-biose (LNB). (PPTX) [file ppat.1007865.s003.pptx]

## Slide 1
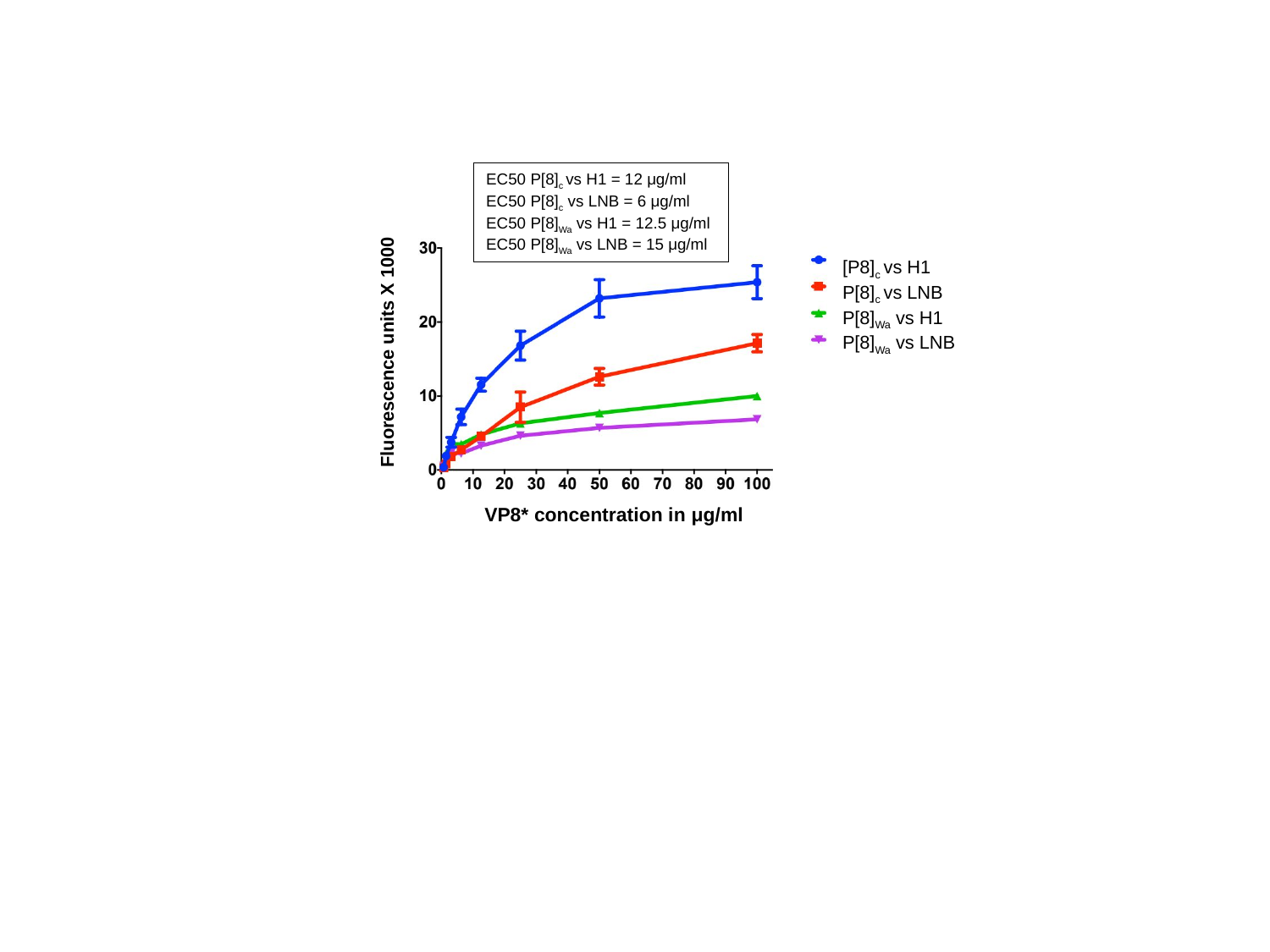

EC50 P[8]c vs H1 = 12 μg/ml
EC50 P[8]c vs LNB = 6 μg/ml
EC50 P[8]Wa vs H1 = 12.5 μg/ml
EC50 P[8]Wa vs LNB = 15 μg/ml
[P8]c vs H1
P[8]c vs LNB
P[8]Wa vs H1
P[8]Wa vs LNB
Fluorescence units X 1000
VP8* concentration in μg/ml
