# Supplementary material for: Unraveling the role of the secretor antigen in human rotavirus attachment to histo-blood group antigens
Source: PLoS Pathog. 2019 Jun 21;15(6):e1007865. doi: 10.1371/journal.ppat.1007865 (PMC6609034; doi:10.1371/journal.ppat.1007865)
Supplement: S5 Fig — a to f Lack of binding of mutants M1 to M3 to the H1 antigen (a, c and e) and to LNB (b, d and f), confirming the functionality of the VP8* binding pocket. g and h show binding and obtained Kda of the M4 mutant (Ile175Val P[8]c) to H1 and LNB, respectively. (PPTX) [file ppat.1007865.s005.pptx]

## Slide 1
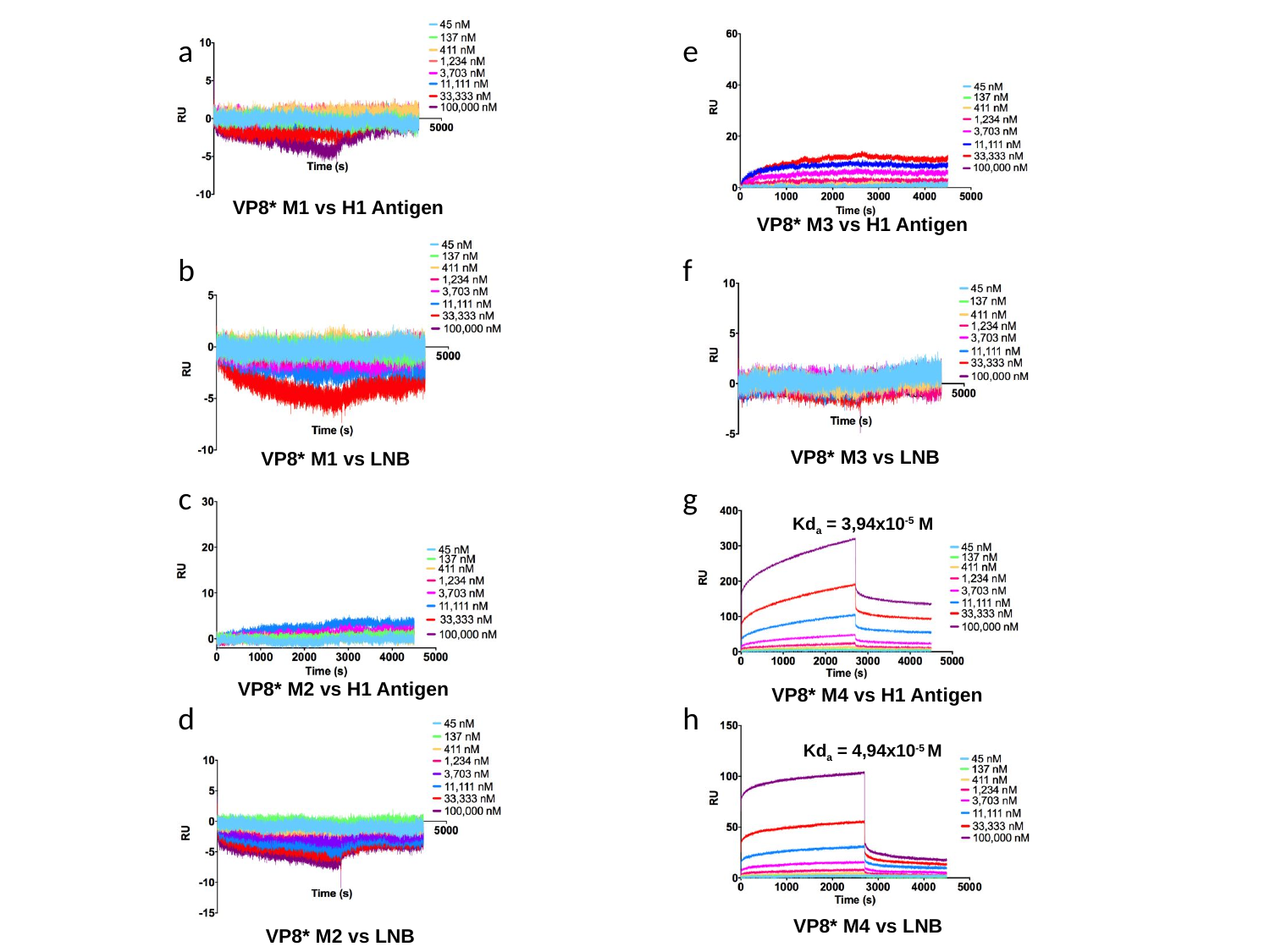

a
e
VP8* M1 vs H1 Antigen
VP8* M3 vs H1 Antigen
b
f
VP8* M3 vs LNB
VP8* M1 vs LNB
c
g
Kda = 3,94x10^-5^ M
VP8* M2 vs H1 Antigen
VP8* M4 vs H1 Antigen
d
h
Kda = 4,94x10^-5^ M
VP8* M4 vs LNB
VP8* M2 vs LNB
