# Supplementary material for: Unraveling the role of the secretor antigen in human rotavirus attachment to histo-blood group antigens
Source: PLoS Pathog. 2019 Jun 21;15(6):e1007865. doi: 10.1371/journal.ppat.1007865 (PMC6609034; doi:10.1371/journal.ppat.1007865)
Supplement: S1 Table — All biotinylated oligosaccharides were purchased from Glyconz. (PPTX) [file ppat.1007865.s009.pptx]

## Slide 1
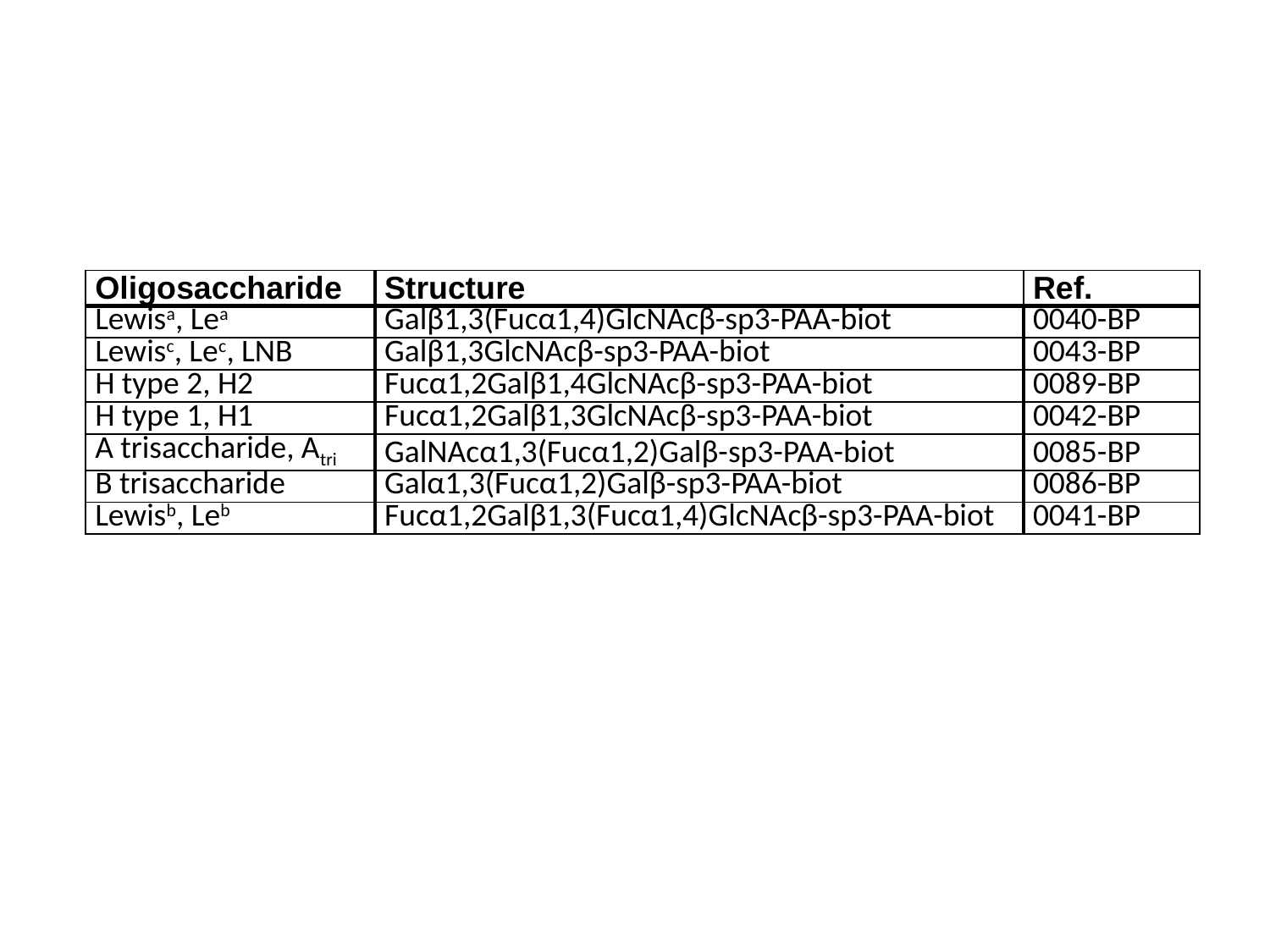

| Oligosaccharide | Structure | Ref. |
| --- | --- | --- |
| Lewisa, Lea | Galβ1,3(Fucα1,4)GlcNAcβ-sp3-PAA-biot | 0040-BP |
| Lewisc, Lec, LNB | Galβ1,3GlcNAcβ-sp3-PAA-biot | 0043-BP |
| H type 2, H2 | Fucα1,2Galβ1,4GlcNAcβ-sp3-PAA-biot | 0089-BP |
| H type 1, H1 | Fucα1,2Galβ1,3GlcNAcβ-sp3-PAA-biot | 0042-BP |
| A trisaccharide, Atri | GalNAcα1,3(Fucα1,2)Galβ-sp3-PAA-biot | 0085-BP |
| B trisaccharide | Galα1,3(Fucα1,2)Galβ-sp3-PAA-biot | 0086-BP |
| Lewisb, Leb | Fucα1,2Galβ1,3(Fucα1,4)GlcNAcβ-sp3-PAA-biot | 0041-BP |
