# Supplementary material for: Unraveling the role of the secretor antigen in human rotavirus attachment to histo-blood group antigens
Source: PLoS Pathog. 2019 Jun 21;15(6):e1007865. doi: 10.1371/journal.ppat.1007865 (PMC6609034; doi:10.1371/journal.ppat.1007865)
Supplement: S2 Table — (PPTX) [file ppat.1007865.s010.pptx]

## Slide 1
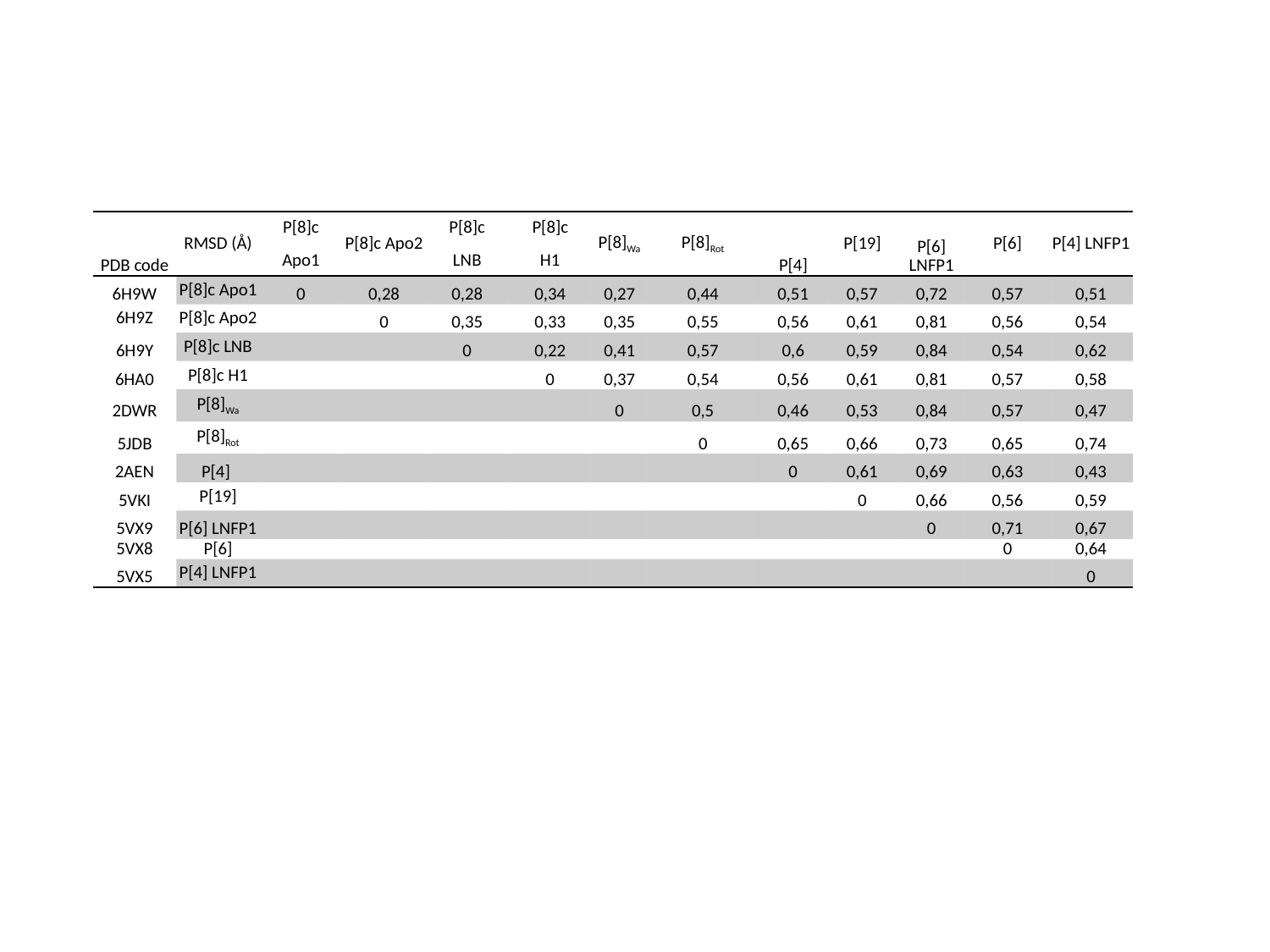

| PDB code | RMSD (Å) | P[8]c Apo1 | P[8]c Apo2 | P[8]c LNB | P[8]c H1 | P[8]Wa | P[8]Rot | P[4] | P[19] | P[6] LNFP1 | P[6] | P[4] LNFP1 |
| --- | --- | --- | --- | --- | --- | --- | --- | --- | --- | --- | --- | --- |
| 6H9W | P[8]c Apo1 | 0 | 0,28 | 0,28 | 0,34 | 0,27 | 0,44 | 0,51 | 0,57 | 0,72 | 0,57 | 0,51 |
| 6H9Z | P[8]c Apo2 | | 0 | 0,35 | 0,33 | 0,35 | 0,55 | 0,56 | 0,61 | 0,81 | 0,56 | 0,54 |
| 6H9Y | P[8]c LNB | | | 0 | 0,22 | 0,41 | 0,57 | 0,6 | 0,59 | 0,84 | 0,54 | 0,62 |
| 6HA0 | P[8]c H1 | | | | 0 | 0,37 | 0,54 | 0,56 | 0,61 | 0,81 | 0,57 | 0,58 |
| 2DWR | P[8]Wa | | | | | 0 | 0,5 | 0,46 | 0,53 | 0,84 | 0,57 | 0,47 |
| 5JDB | P[8]Rot | | | | | | 0 | 0,65 | 0,66 | 0,73 | 0,65 | 0,74 |
| 2AEN | P[4] | | | | | | | 0 | 0,61 | 0,69 | 0,63 | 0,43 |
| 5VKI | P[19] | | | | | | | | 0 | 0,66 | 0,56 | 0,59 |
| 5VX9 | P[6] LNFP1 | | | | | | | | | 0 | 0,71 | 0,67 |
| 5VX8 | P[6] | | | | | | | | | | 0 | 0,64 |
| 5VX5 | P[4] LNFP1 | | | | | | | | | | | 0 |
