# Supplementary material for: Unraveling the role of the secretor antigen in human rotavirus attachment to histo-blood group antigens
Source: PLoS Pathog. 2019 Jun 21;15(6):e1007865. doi: 10.1371/journal.ppat.1007865 (PMC6609034; doi:10.1371/journal.ppat.1007865)
Supplement: S5 Table — BamHI restriction site is underlined. (PPTX) [file ppat.1007865.s013.pptx]

## Slide 1
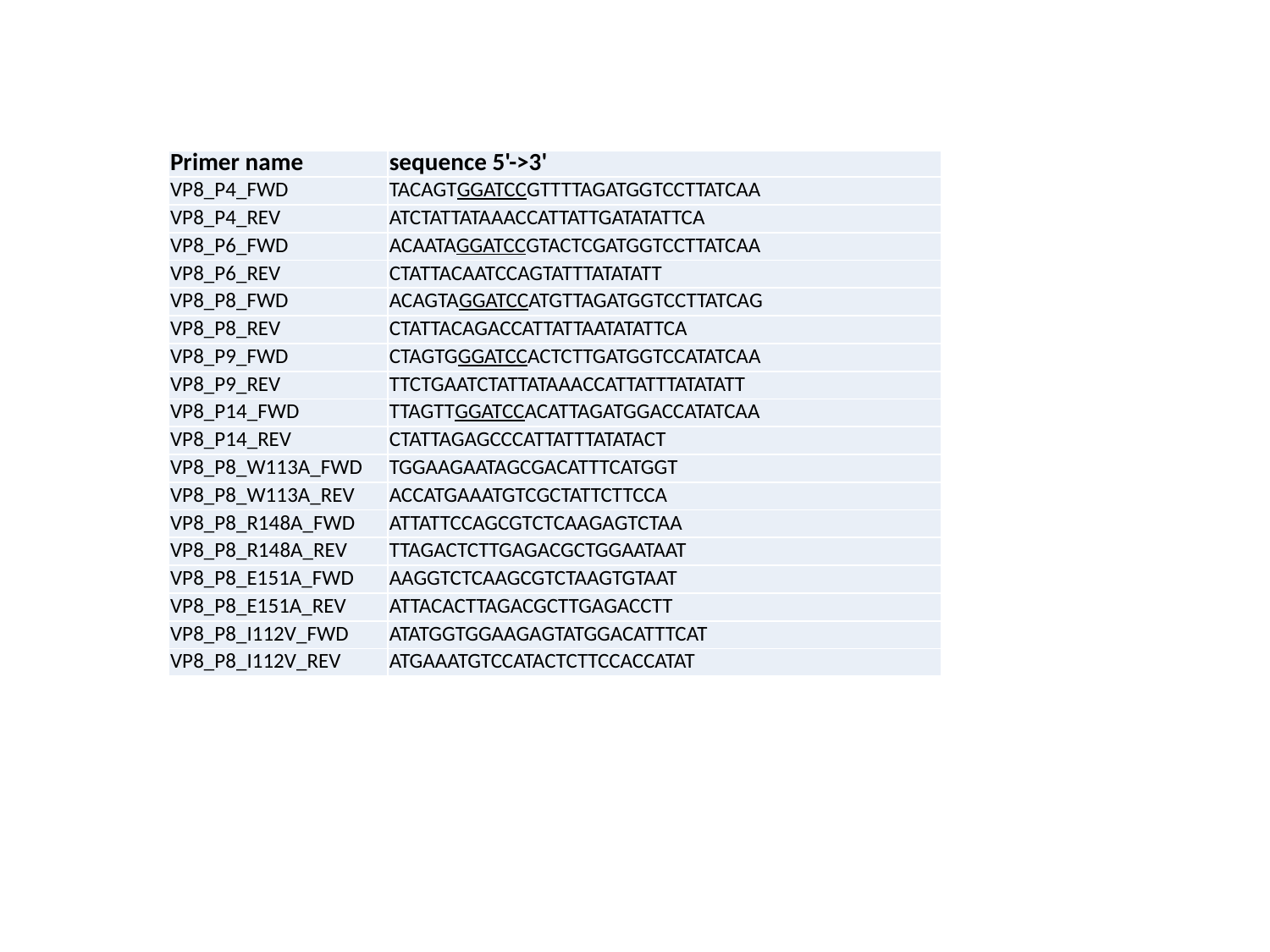

| Primer name | sequence 5'->3' |
| --- | --- |
| VP8\_P4\_FWD | TACAGTGGATCCGTTTTAGATGGTCCTTATCAA |
| VP8\_P4\_REV | ATCTATTATAAACCATTATTGATATATTCA |
| VP8\_P6\_FWD | ACAATAGGATCCGTACTCGATGGTCCTTATCAA |
| VP8\_P6\_REV | CTATTACAATCCAGTATTTATATATT |
| VP8\_P8\_FWD | ACAGTAGGATCCATGTTAGATGGTCCTTATCAG |
| VP8\_P8\_REV | CTATTACAGACCATTATTAATATATTCA |
| VP8\_P9\_FWD | CTAGTGGGATCCACTCTTGATGGTCCATATCAA |
| VP8\_P9\_REV | TTCTGAATCTATTATAAACCATTATTTATATATT |
| VP8\_P14\_FWD | TTAGTTGGATCCACATTAGATGGACCATATCAA |
| VP8\_P14\_REV | CTATTAGAGCCCATTATTTATATACT |
| VP8\_P8\_W113A\_FWD | TGGAAGAATAGCGACATTTCATGGT |
| VP8\_P8\_W113A\_REV | ACCATGAAATGTCGCTATTCTTCCA |
| VP8\_P8\_R148A\_FWD | ATTATTCCAGCGTCTCAAGAGTCTAA |
| VP8\_P8\_R148A\_REV | TTAGACTCTTGAGACGCTGGAATAAT |
| VP8\_P8\_E151A\_FWD | AAGGTCTCAAGCGTCTAAGTGTAAT |
| VP8\_P8\_E151A\_REV | ATTACACTTAGACGCTTGAGACCTT |
| VP8\_P8\_I112V\_FWD | ATATGGTGGAAGAGTATGGACATTTCAT |
| VP8\_P8\_I112V\_REV | ATGAAATGTCCATACTCTTCCACCATAT |
