# Supplementary material for: Unraveling the role of the secretor antigen in human rotavirus attachment to histo-blood group antigens
Source: PLoS Pathog. 2019 Jun 21;15(6):e1007865. doi: 10.1371/journal.ppat.1007865 (PMC6609034; doi:10.1371/journal.ppat.1007865)
Supplement: S6 Table — (PPTX) [file ppat.1007865.s014.pptx]

## Slide 1
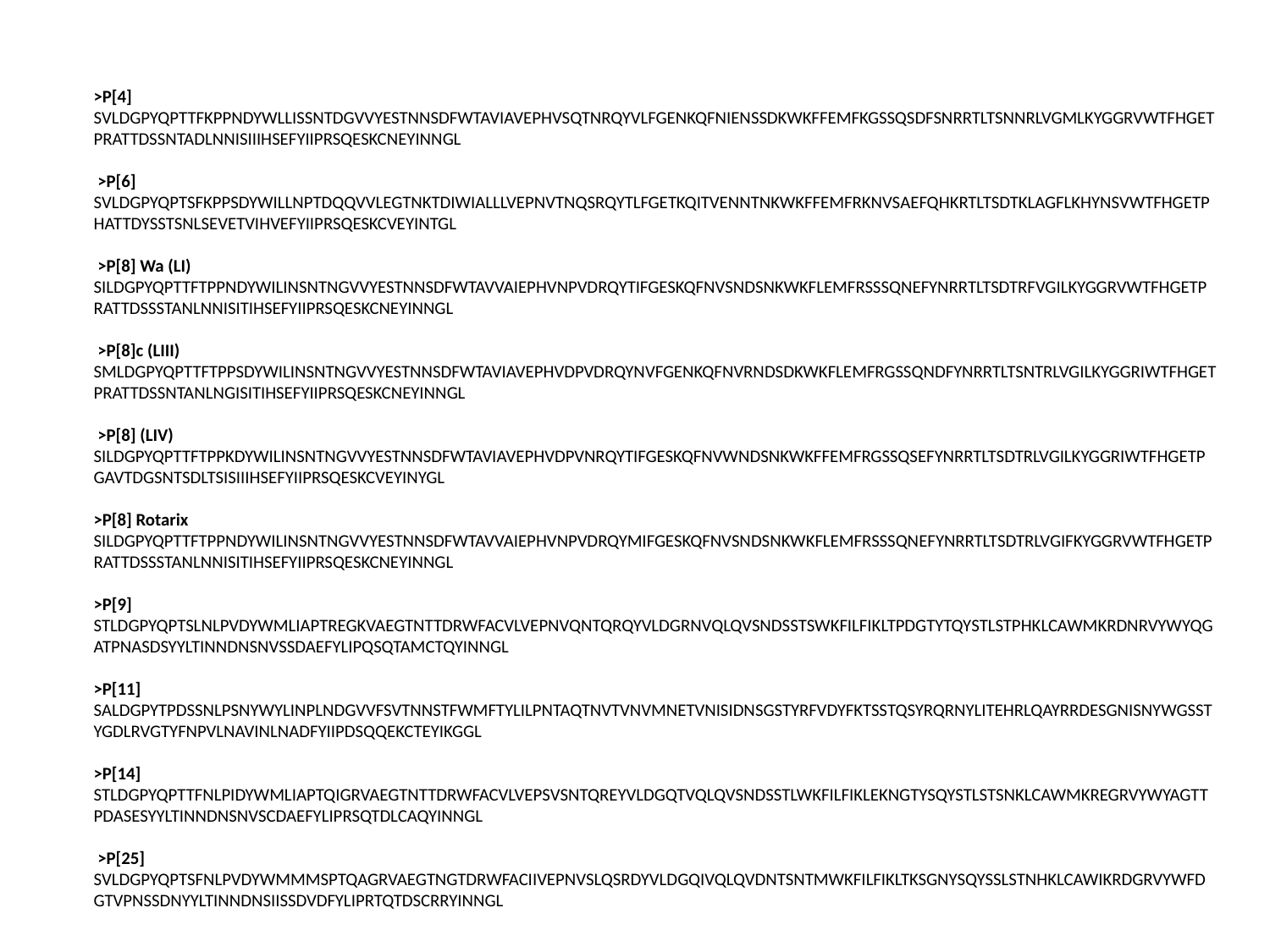

>P[4]
SVLDGPYQPTTFKPPNDYWLLISSNTDGVVYESTNNSDFWTAVIAVEPHVSQTNRQYVLFGENKQFNIENSSDKWKFFEMFKGSSQSDFSNRRTLTSNNRLVGMLKYGGRVWTFHGETPRATTDSSNTADLNNISIIIHSEFYIIPRSQESKCNEYINNGL
 >P[6]
SVLDGPYQPTSFKPPSDYWILLNPTDQQVVLEGTNKTDIWIALLLVEPNVTNQSRQYTLFGETKQITVENNTNKWKFFEMFRKNVSAEFQHKRTLTSDTKLAGFLKHYNSVWTFHGETPHATTDYSSTSNLSEVETVIHVEFYIIPRSQESKCVEYINTGL
 >P[8] Wa (LI)
SILDGPYQPTTFTPPNDYWILINSNTNGVVYESTNNSDFWTAVVAIEPHVNPVDRQYTIFGESKQFNVSNDSNKWKFLEMFRSSSQNEFYNRRTLTSDTRFVGILKYGGRVWTFHGETPRATTDSSSTANLNNISITIHSEFYIIPRSQESKCNEYINNGL
 >P[8]c (LIII)
SMLDGPYQPTTFTPPSDYWILINSNTNGVVYESTNNSDFWTAVIAVEPHVDPVDRQYNVFGENKQFNVRNDSDKWKFLEMFRGSSQNDFYNRRTLTSNTRLVGILKYGGRIWTFHGETPRATTDSSNTANLNGISITIHSEFYIIPRSQESKCNEYINNGL
 >P[8] (LIV)
SILDGPYQPTTFTPPKDYWILINSNTNGVVYESTNNSDFWTAVIAVEPHVDPVNRQYTIFGESKQFNVWNDSNKWKFFEMFRGSSQSEFYNRRTLTSDTRLVGILKYGGRIWTFHGETPGAVTDGSNTSDLTSISIIIHSEFYIIPRSQESKCVEYINYGL
>P[8] Rotarix
SILDGPYQPTTFTPPNDYWILINSNTNGVVYESTNNSDFWTAVVAIEPHVNPVDRQYMIFGESKQFNVSNDSNKWKFLEMFRSSSQNEFYNRRTLTSDTRLVGIFKYGGRVWTFHGETPRATTDSSSTANLNNISITIHSEFYIIPRSQESKCNEYINNGL
>P[9]
STLDGPYQPTSLNLPVDYWMLIAPTREGKVAEGTNTTDRWFACVLVEPNVQNTQRQYVLDGRNVQLQVSNDSSTSWKFILFIKLTPDGTYTQYSTLSTPHKLCAWMKRDNRVYWYQGATPNASDSYYLTINNDNSNVSSDAEFYLIPQSQTAMCTQYINNGL
>P[11]
SALDGPYTPDSSNLPSNYWYLINPLNDGVVFSVTNNSTFWMFTYLILPNTAQTNVTVNVMNETVNISIDNSGSTYRFVDYFKTSSTQSYRQRNYLITEHRLQAYRRDESGNISNYWGSSTYGDLRVGTYFNPVLNAVINLNADFYIIPDSQQEKCTEYIKGGL
>P[14]
STLDGPYQPTTFNLPIDYWMLIAPTQIGRVAEGTNTTDRWFACVLVEPSVSNTQREYVLDGQTVQLQVSNDSSTLWKFILFIKLEKNGTYSQYSTLSTSNKLCAWMKREGRVYWYAGTTPDASESYYLTINNDNSNVSCDAEFYLIPRSQTDLCAQYINNGL
 >P[25]
SVLDGPYQPTSFNLPVDYWMMMSPTQAGRVAEGTNGTDRWFACIIVEPNVSLQSRDYVLDGQIVQLQVDNTSNTMWKFILFIKLTKSGNYSQYSSLSTNHKLCAWIKRDGRVYWFDGTVPNSSDNYYLTINNDNSIISSDVDFYLIPRTQTDSCRRYINNGL
